# Systematically Improvable and Locality Accelerated Enzymatic Reactivity Modeling: Toward Chemical Accuracy at Affordable Cost

**DOI:** 10.1021/acs.jctc.5c02128

**Published:** 2026-03-24

**Authors:** Dénes Berta, József Csóka, Gyula Samu, Péter R. Nagy

**Affiliations:** † Department of Physical Chemistry and Materials Science, Faculty of Chemical Technology and Biotechnology, 61810 Budapest University of Technology and Economics, Müegyetem rkp. 3., H-1111 Budapest, Hungary; ‡ HUN-REN−BME Quantum Chemistry Research Group, Müegyetem rkp. 3., H-1111 Budapest, Hungary; § MTA−BME Lendület Quantum Chemistry Research Group, Müegyetem rkp. 3., H-1111 Budapest, Hungary

## Abstract

Quantum mechanics/molecular mechanics (QM/MM) is the cornerstone of computational enzymology. Herein, we address an outstanding challenge in QM/MM, namely, simultaneous access to accurate QM methodology and a large QM subsystem at an affordable computational cost. First, local natural orbital (LNO)-based CCSD­(T) is employed for chemically accurate energetics and as a reference for choosing density functional theory (DFT) models. Next, reliable hybrid DFT methods are selected, with large QM subsystem selections suitable also for reaction barriers. Then, quantum embedding, especially accelerated via our recent local embedded subsystem (LESS) approach, is used to reduce the cost of DFT calculations to a few core hours, even with large QM sizes up to ca. 400 QM atoms. By combining these advanced methods, we propose a Locality Accelerated (by LESS and LNO) and Systematically Improvable (LASI) scheme for QM/MM simulations. It benefits from the strengths of a converged QM size in its DFT component, affordability for many configurations via quantum embedding, and, if needed, CCSD­(T) accuracy for energetics. The protocol is validated through the study of challenging, representative, and clinically relevant enzyme-catalyzed phosphate hydrolysis. Based on these results, we establish generally applicable guidelines to set up the components of the LASI protocol. The flexibility and affordability of LASI, both in large-scale QM and QM/MM contexts, make it broadly applicable for the predictive computational description of enzyme reactivity and beyond.

## Graphical abstract

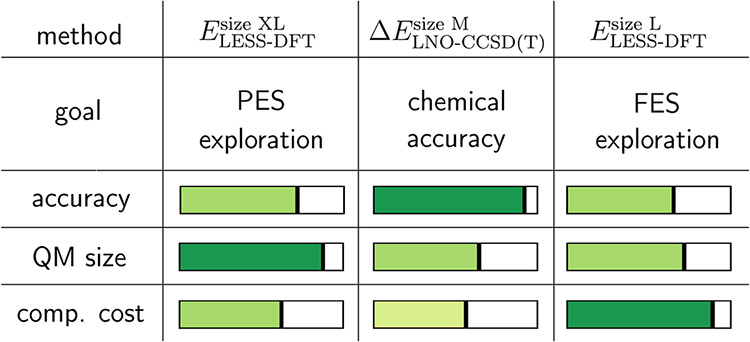

## 1 Introduction

The computational description of enzyme catalysis provides a powerful framework for elucidating complex biochemical reactions. Hybrid quantum mechanics/molecular mechanics (QM/MM) has emerged as the *de facto* methodology for simulating reactions in biomolecular environments, becoming essential in mechanistic studies and in understanding interactions driving drug design.^1–3^ The comparison of experimental and computational properties hinges on the calculation of (relative) free energies, as these thermodynamic quantities are the basis for robust validation.^4^ A validated simulation protocol transitions from a descriptive tool into a predictive one capable of guiding the rational design of new molecules or materials.

Obtaining accurate kinetic and thermodynamic properties necessitates a careful compromise between multiple coupled aspects. Here, we focus on three aspects of central importance: (A1) the accuracy of the QM method, (A2) the size of the subsystem treated by the QM method, and (A3) the exhaustiveness of the statistical sampling technique.^5^ Our objective is to advance QM/MM methodologies, well-converged in QM accuracy (A1) and QM size (A2), in a way that when combined with enhanced sampling applications (A3), free energy simulations will still be affordable. Although the choice of each simulation parameter introduces inherent and coupled uncertainties, recent advances enable us to approach them via systematically improvable methods. The convergence of properties with regard to a single aspect (either QM accuracy, QM size, or sampling) has been established in various cases; however, combining the individually converged levels remains prohibitively expensive.

Regarding A1, gold-standard, wave-function-based methods enable chemical accuracy (ca. 1 kcal/mol). These are becoming increasingly affordable by themselves and can also provide references for selecting reliable, e.g., hybrid level density functional theory (DFT) models. Concurrently, for A2, many kinds of computed properties exhibit slow convergence with respect to the QM region size. In turn, calculations at such converged QM sizes have been limited to a small number of structures with hybrid DFT. On top of these challenges, the rigorous demands of statistical thermodynamics necessitate even more compromise in A1–A2. Specifically, the high computational cost of extensive sampling, which is required for free energy convergence, often forces practical compromises on the DFT model, basis set, and the QM region size, thereby introducing limitations in accuracy.

Coupled cluster (CC) with single, double, and perturbative triple excitation [CCSD­(T)] methods^6^ represent the gold standard regarding the accuracy (A1) of quantum methods, while the best and well-selected DFT functionals may also approach chemical accuracy for a specific reaction. Local-correlation-based CCSD­(T) calculations, such as our local natural orbital (LNO) approach, are now accessible relatively routinely for systems comprising several hundred atoms, providing excellent energetic predictions.^7^ However, CCSD­(T) forces are not yet available for large molecules, limiting their use in free energy sampling. Using such high-level references, accurate DFT functionals can be rationally selected for potential energy optimizations, enabling identification of key transition and intermediate states.^8^

The convergence of computed properties with respect to the size of the QM region is a critical consideration (A2).^9,10^ Enlarging the QM region results in more complete incorporation of quantum interactions, like electronic polarization, charge transfer, long-range dispersion, and their coupling, which enhances accuracy but increases computational demand steeply. Practical limitations arise from the computational cost, thus requiring careful selection of the QM region that balances capturing the essential chemistry with tractability. Studies on the convergence of calculated properties suggest that QM regions may often need to contain several hundreds of atoms for properties to converge.^11–15^ However, in practice, selections are often compromised due to the computational cost of the chosen QM methodology, and stop short of size-convergence (A2) at around 100 QM atoms.^5^

Free energy methods to characterize reaction thermodynamics in QM/MM contexts typically involve sampling techniques (A3) to compute measurable properties through statistical means.^16^ For reactions, this is further complicated by regions in the free energy landscape that are visited with a low probability (e.g., toward transition states). Simulations thus rely on enhanced sampling methods, like umbrella sampling, metadynamics, thermodynamic integration or the string method.^17,18^ Despite their successes, these approaches face limitations because convergence requires exhaustive sampling of the high-dimensional phase space, and the computational cost of ab initio QM/MM calculations restricts the accessible simulation time scales and system sizes.^19,20^ Consequently, their combination with accurate QM levels of theory and sufficiently large QM regions are hampered.^21^ The common choices are for converged statistics (A3), which most often rely on classical, semiempirical, or recently also machine learned force field methods.^22–26^

We demonstrate these limitations and the capabilities of our proposed approach to overcome them using the example of GTP hydrolysis catalyzed by the extensively studied small GTPase Ras. The clinical relevance of Ras is in its high rate of mutation in cancer cell lines, which is also associated with poor prognosis.^27,28^ GTPases cleave the terminal phosphate group of the anhydride chain in GTP (Figure 1). Breaking or forming of P–O bonds is a common process in biochemical reactions, as phosphates are an essential building block of living organisms.^29,30^ The mechanism of Ras GTPase has been extensively studied.^31^ Building on this, we use five stationary points (Figure 1) to represent the general base-catalyzed mechanism published in ref 20. The heavily polarized active site is challenging for frequently used DFT methods,^32–34^ especially because it includes numerous explicitly charged residues, such as GTP^4–^/GDP^3–^ coordinated Mg^2+^ ion. The S_N_2 character of the phosphate cleavage poses further difficulty for practical density functional approximations. The associated S_N_2 TS is known to require at least hybrid DFT, driving the cost of QM/MM calculations. The phosphate cleavage is followed by a proton transfer step, making this a representative real-life system to study the accuracy and limitations of QM/MM modeling describing phosphate biochemistry.^31^

**Figure 1:**
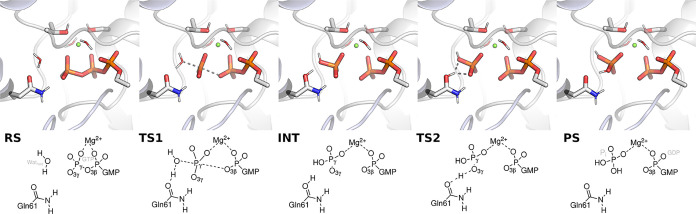
Stationary points along the Gln61-assisted GTP hydrolysis mechanism of Ras GTPase: RS: reactant state, TS1: transition state 1, INT: intermediate, TS2: transition state 2, PS: product state. Forming and breaking bonds are depicted in gray dashes. The guanosine of the GTP and aliphatic hydrogens are omitted for clarity.

Herein, we propose improvements to QM/MM approaches by extending the use of cutting-edge computational techniques to model biochemical reactivity. We specifically focus on the coupling and systematic convergence of QM methods and QM region selection toward reaching chemical accuracy in A1 and A2. Moreover, we do so by also working with the computational budget restraints required for enhanced sampling applications (A3).

To that end, the following advances and demonstrations are presented: The convergence of reaction barriers and energies is studied against QM region sizes up to several hundred atoms. From this, we identify general selection principles that help achieve converged results with fewer resources. We demonstrate the applicability of well-converged LNO-CCSD­(T) calculations for QM regions of up to about 400 atoms with accessible resources. Such reference values are used here to select robust and accurate DFT methodologies, including a sufficiently large basis set for the studied phosphate chemistry. Crucially, we study the connection between convergence with the QM method and the region size to select the appropriate model parameters. We compare multiple ways of accelerating DFT calculations, of which our recently developed local embedded subsystem (LESS) framework stands out.^35^ In LESS, we start with a quantum mechanical embedding approach, which in the present context entails hybrid DFT for the subsystem involved in chemical change and GGA DFT for an extended protein environment that still cannot be accurately modeled via MM. LESS exploits that the high-level hybrid DFT computation can be greatly accelerated by a strategic selection of not only the high-level atoms and occupied orbitals (like in previous quantum embedding variations) but also the atomic and density fitting basis functions.^35^ We demonstrated recently that LESS practically achieves hybrid DFT accuracy at about twice the cost of GGA DFT.^35^ Our approach bears two recurring characteristics: first, it relies on performance acceleration via exploiting the locality of the chemical process. LESS achieves acceleration for accurate DFT calculations, while the LNO-based CC provides affordable chemical accuracy. Second, it builds on systematic improvability in terms of the employed local and embedding approximations as well as the converging series of basis set and QM subsystem size choices. Thus, besides useful defaults, we present a process to select accurate and efficient methodologies tailored for different simulation goals.

## 2 Methods

### 2.1 Locality Accelerated, Systematically Improvable (LASI) Enzymatic Thermochemistry

We propose a relatively free energy scheme, LASI for short, that is conscious of both the systematic improvability and affordability of different methodologies, aiming at the best possible accuracy and transferability allowed by state-of-the-art methods. For a given system, we denote the most accurate model that is computationally compatible (e.g., can compute gradients) and fast enough to be used jointly with sampling (S) techniques as DFT_S_. In other words, DFT_S_ embodies the optimum for statistical measures and consequently the calculation of entropic contributions. Hence, free energy simulations comprising tens of thousands of points have to be possible on the level of DFT_S_, from which entropic contributions are calculated. If required, moving toward the model sizes and/or QM levels that are closer to the converged results, we propose to find the most quantitatively accurate method DFT_E_. It can be applied to identify reaction paths still requiring the evaluation of atomic forces at hundreds of points, but this DFT_E_ model mainly operates on the PES due to its higher cost. DFT_E_ puts the focus on A1 and A2 optimization, as it is intended for the evaluation of a smaller number of configurations. For a more limited number of structures, such as the comparison of stationary points or a discretely sampled reaction path, converged LNO-CCSD­(T) is affordable. Using this as a correction to DFT_E_ energies (ΔΔ*E*_CC_) improves the energetics toward chemical accuracy.

Therefore, the proposed free energy scheme (with all terms calculated in a QM/MM manner as electrostatically embedded into an MM environment) is as follows

1 $$\Delta G=\Delta E_{{\mathrm{DFT}}_{E}}+\left[\Delta \Delta E_{\mathrm{CC}}\right]-T\Delta S_{{\mathrm{DFT}}_{S}}$$

If it is affordable, it is simpler to use the same DFT_S_ and DFT_E_ model. We keep them separated, as DFT_E_ might still be expensive for practical free energy simulations at the level of sufficiently converged DFT level, QM regions, and basis sets. Next, we overview the theoretical and technical developments that we leverage in determining the specific settings for the LASI components DFT_E_, DFT_S_, and *Δ*Δ*E*_CC_, while keeping their respective cost requirements in mind.

### 2.2 Local Coupled-Cluster Calculations

For gold-standard references, well-converged CCSD­(T) electronic energies are computed, using the efficient local natural orbital (LNO)^7,36–39^ method of the Mrcc program package.^40–42^ Extrapolation toward the complete basis set (CBS) and the local approximation free (LAF)^38^ limits of LNO-CCSD­(T) are both performed. Therefore, the reference composite energy scheme is written as follows:

2 $$E_{N-\mathrm{TLNO}-\mathrm{CCSD}\left(T\right)}^{\mathrm{CBS}\left(X,X+1\right)}=E_{\mathrm{Normal}}^{\mathrm{CBS}\left(X,X+1\right)}+E_{N-T}^{X}-E_{\mathrm{Normal}}^{X}$$

where *X* stands for the cardinal number in the used atomic orbital (AO) basis sets, N and T refer to the *Normal* and *Tight* local-correlation thresholds, and N-T denotes the extrapolation from these toward the LAF limit. This protocol in eq 2 was found accurate and affordable across a broad range of chemical applications, and its validity can always be checked system-specifically, like we do below.^7^ The basis set convergence is further accelerated by adding a density-based basis set correction (DBBSC) to the LNO-CCSD­(T) values.^39^ DBBSC provides a short-range DFT-based correction for the incompleteness of the specific finite AO basis set used for wave-function methods. This is achieved via a finite basis electron–electron interaction formulation that uses the density of the system.^43,44^ Thus, DBBSC corrects for the short-range finite basis error that is dominantly responsible for the slow basis set convergence of wave-function methods. Our LNO-based DBBSC implementation provides asymptotically linear-scaling and very low overhead for the rate-determining step of DBBSC, i.e., the range-separation function of the DFT correction that adapts to the spatial inhomogeneity of the basis-set incompleteness.^39^

We establish the convergence of relative energies regarding both basis sets and thresholds on smaller QM regions and carry out LNO-CCSD­(T)/CBS calculations up to the QM size used in DFT_E_ calculations (see Figure 4 and section S3 for more details). The accuracy of such references is excellent for benchmarking purposes, and their effectiveness enables their usage in eq 1 for 10s–100s of configurations along the reaction coordinate. Moreover, the approach fits into our overall strategy, as the basis set, LNO threshold, and the QM size can all be systematically converged to chemical accuracy. An additional benefit is that robust error estimates can be assigned to monitor the level of convergence.^7,38^

### 2.3 Speeding up DFT Calculations via Huzinaga Quantum Embedding

When modeling reactions, lower rung DFT functionals, like GGAs or mGGAs, are known to overstabilize some transition states, e.g., due to self-interaction error (SIE), like for **TS1** in Figure 1.^8^ The chosen enzymatic reaction, representing features typical in biochemistry, also involves multiple bond breaking/forming events. The environment is highly polarized by numerous explicitly charged moieties, posing further challenges for DFT models. Therefore, hybrid or higher-level DFT methods are required, setting a computational bottleneck in QM/MM, especially limiting free energy simulations.

To speed up the DFT calculation, we advance the utility of quantum embedding methods for enzyme modeling.^45–47^ Here, in addition to QM/MM, we split the QM calculation into two QM layers. This enables fast evaluation by limiting the expensive but accurate high-level (HL) QM description to the chemically active subsystem (A). Among the numerous QM-QM embedding schemes, our implementation is an improved variant of projection-based embedding formalisms,^48^ using the Huzinaga equation.^35,49^ The total energy of the QM-QM system is written as

$$E=E_{\mathrm{LL}}\left[D\right]-E_{\mathrm{LL}}\left[D^{A}\right]+E_{\mathrm{HL}}\left[{\mathrm{D̃}}^{A}\right]+\mathrm{Tr}\;\left\{\left({\mathrm{D̃}}^{A}-D^{A}\right)\frac{\partial E_{\mathrm{LL}-\mathrm{HL}}}{\partial D^{A}}\right\}$$ 3

where LL and HL stand for the low- and high-level models; ***D*** and ***D*** ^*A*^ refer to the LL density of the full and the active subsystem, respectively, while ***D* ~**^*A*^ denotes the HL density calculated for the active subsystem. The last term of eq 3 is a first-order correction in the Huzinaga-embedding energy using the active subsystem density difference and

4 $$E_{\mathrm{LL}-\mathrm{HL}}=E_{\mathrm{LL}}\left[D\right]-E_{\mathrm{LL}}\left[D^{A}\right]+E_{\mathrm{HL}}\left[{\mathrm{D̃}}^{A}\right]$$

The separation of the HL and LL subsystems creates an opportunity to choose a more efficient DFT method and a smaller basis set for the LL environment while retaining the HL description for local processes in the active layer. In practice, first, the LL calculation is performed for the entire QM system, and then the occupied molecular orbitals (MOs) are localized. The MOs residing on the subsystem atoms are selected as active MOs (orange halos in Figure 2), based on the Mulliken population analysis^48,49^ or via other methods.^50,51^ From the practical perspective, the user only needs to select the active atom list; the MO selection is automated. Then, the high-level MOs are optimized in an embedding potential with the HL SCF method, while the environment MOs are kept frozen.

**Figure 2:**
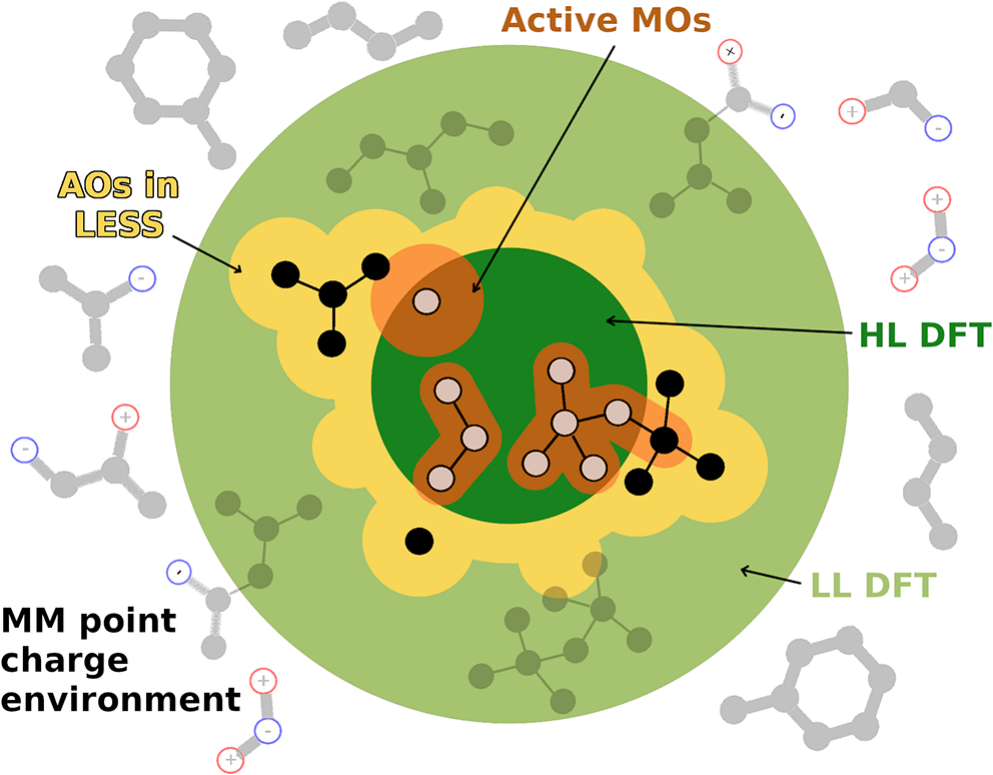
Schematic representation of the LESS framework inside an electrostatically embedded MM environment (gray balls and sticks with charges). The high-level calculation of the active subsystem (dark green) comprises the active atoms (hollow balls), only utilizes the MOs (orange spheres), and the AO and auxiliary functions (yellow halo). The low-level calculation of the full QM system is depicted in light green.

To further accelerate the HL computation, we have recently introduced the local embedded subsystem (LESS) framework, which decreases the size of the AO basis set as well as the auxiliary basis set when density fitting (DF) is used (yellow halos in Figure 2). This yields an asymptotically constant cost for the HL part at the limit of a much larger environment around a finite active system. Consequently, only those AO and DF basis functions are used for the HL calculation of the embedded subsystem that are important to accurately expand the active MOs and Huzinaga-embedding quantities needed for the HL part. Therefore, the LESS approximations can drastically reduce the number of DF integrals, which in turn can be stored in memory (in-core) for the HL calculation. The calculation of analytical gradients is already available in our Huzinaga-type embedding implementation.^52^ We are actively working on extending that to the LESS scheme to exploit the associated speed-up in optimizations and MD simulations.

We previously demonstrated that LESS is universally applicable for various catalytic (homogeneous, surface, and enzymatic) reactions to a similar effect: accuracy of a QM_HL_ method obtained at the cost of 2–3 QM_LL_ calculations due to the 50–100-fold acceleration of the QM_HL_ part.^35^ Based on those diverse cases, reasonable defaults are also available for basis truncation algorithms. Importantly, one can always systematically converge to the QM_HL_ results by improving the QM_HL_ region definition as well as the AO or DF basis selection thresholds.

An alternative for speeding up hybrid DFT calculations is our local DF (LDF) algorithm.^53–55^ Here, a set of atoms (a domain) is automatically selected for each localized MO, so that the auxiliary functions centered on the selected atoms are used in the exchange term for DF. The LDF approach can significantly speed up the hybrid DFT calculation without relying on embedding. The main difference is that the HF exchange contribution is evaluated for all MOs in LDF, while with LESS Huzinaga embedding, the HF exchange evaluation is restricted to the reactive regions.

### 2.4 Selection of the QM Region in QM/MM

In an enzymatic environment, the definition of the QM region of QM/MM will inevitably result in cutting covalent bonds, invoking treatment of free valences by link atoms or localized single electron orbitals.^56,57^ The general advice is to make cuts at apolar single bonds, typically carbon–carbon, whenever possible.^58^ The immediate chemical environment of a reaction is generally straightforward from the reaction in question and from mechanistic ideas. In the context of a reaction, especially with multiple elementary steps, the set of important residues in a product state is not necessarily identical to that of the reactant state. Consequently, with a preliminary reaction path, selections can be made for multiple structures (e.g., reactant, product states), then the union of those could be used in downstream simulations.^59^ Including the reactive moieties and their close contacts often yield a QM region around 100 atoms.^60^ Due to the associated cost of at least hybrid DFT, many studies do not exceed this consideration, while key energetic properties may not be converged to chemical accuracy.

While there are algorithms proposed to aid the definition of QM regions, e.g., based on electrostatics,^61,62^ forces^63^ or contact maps,^64^ the QM region selection of an arbitrary active site does not have a universally accepted technique.^57^ Selection methods are also developed for building QM cluster models, with the potential to be extended for QM/MM simulations.^65,66^ While in most studies with fewer (50–100) QM atoms, the optimization of the QM atom selection is somewhat simpler, this problem becomes more pressing as one aims at convergence with the QM size and therefore includes hundreds of QM atoms. Namely, the complexity of optimal QM atom list selection rapidly grows with the spatial extension of the QM region, and at the same time, the chemical-knowledge-based selection becomes ambiguous. Therefore, the considerations that current (semi)­automated selection algorithms use for building a QM/MM interface is and will be even more valuable as larger QM selections become more affordable.^67^ Consequently, one may anticipate further focus and progress on automated selection algorithms in the future, especially those applied in high-throughput screening procedures. Notably, if the constructed model is intended for extended reaction path optimization and free energy simulation, then thorough manual optimization of the QM region is justified by the downstream gain in computational cost. In other applications, hand-optimized QM selections may require more time investment than the benefit from the QM/MM time reduction. In such cases, one could more easily afford simplified or automated QM region selector algorithms, because of the acceleration offered by the LESS approach for larger total QM selections.

Here, we use a topologically extended distance-based QM selection algorithm as a starting point for our QM region optimization. The extension starts from a manually selected core atom list, which can be as small as a single atom, making sure that we have an appropriate focus on where the reaction happens. Atoms within a defined radius are then added, unlike in some selection methods, which decide the inclusion of residues as a whole. The thus created atom list is then iteratively extended along the covalent bonds until it encounters a cutable bond. The list of bonds that we allow to be cut at the interface is defined for the conventional protein atom types and can be extended in our implementation. The selection algorithm also removes topologically close link atoms: if the MM-side atom is the same at two cutting events, the QM region is automatically extended, thereby decreasing the number of cut bonds at the QM/MM interface. Another important feature is the option to include multiple structures for the selection, which is ideal for considering geometry changes along a preliminary reaction path. The outlined QM atom selection script and its documentation are openly available on GitHub.^68^

## 3 Results

In this section, we present the convergence of reaction energies and barriers with both the increasing size of the QM region and systematically improved computational methods against gold-standard LNO-CCSD­(T) references. We also study the connection between these convergences, determining model details for different cost levels of the LASI scheme discussed in eq 1. Finally, we analyze the effect of multilayer QM methods on the accuracy of the calculated relative energies. Computational details are listed in Section 6.

### 3.1 Initial Optimization of the QM Atom List

The minimal chemically intuitive QM region from the previous QM/MM studies on the selected reaction was 101 atoms, including link atoms.^20^ Further trimming of this resulted in an unrealistic increase in the barrier height of the second step (Figure 3C, right, green “chemical”). We first considered radial extensions in the range of 3 to 9 Å radius from four reactive atoms: the γ-phosphorus, the oxygen of the nucleophilic water, the oxygen atom of the leaving group and the Mg^2+^ (red halos in Figure 3A). Relative energies of the stationary points converge slowly with radial selections, and convergence within 1 kcal/mol is only reached above 431 QM atoms (blue series in Figures 3C and S1). The convergence of the overall relative QM/MM energies above 400 atoms can also be observed in the slow convergence of the dispersion correction or the disappearance of the relative MM contribution (Figure S2). Interestingly, the two reaction steps exhibit very similar overall barrier heights (Figure 3C inset), which makes determining the rate-determining step difficult. This highlights the need for well-converged results, especially when comparing transition states of different natures; otherwise, one may arrive at qualitatively wrong conclusions regarding the mechanistic details.

**Figure 3:**
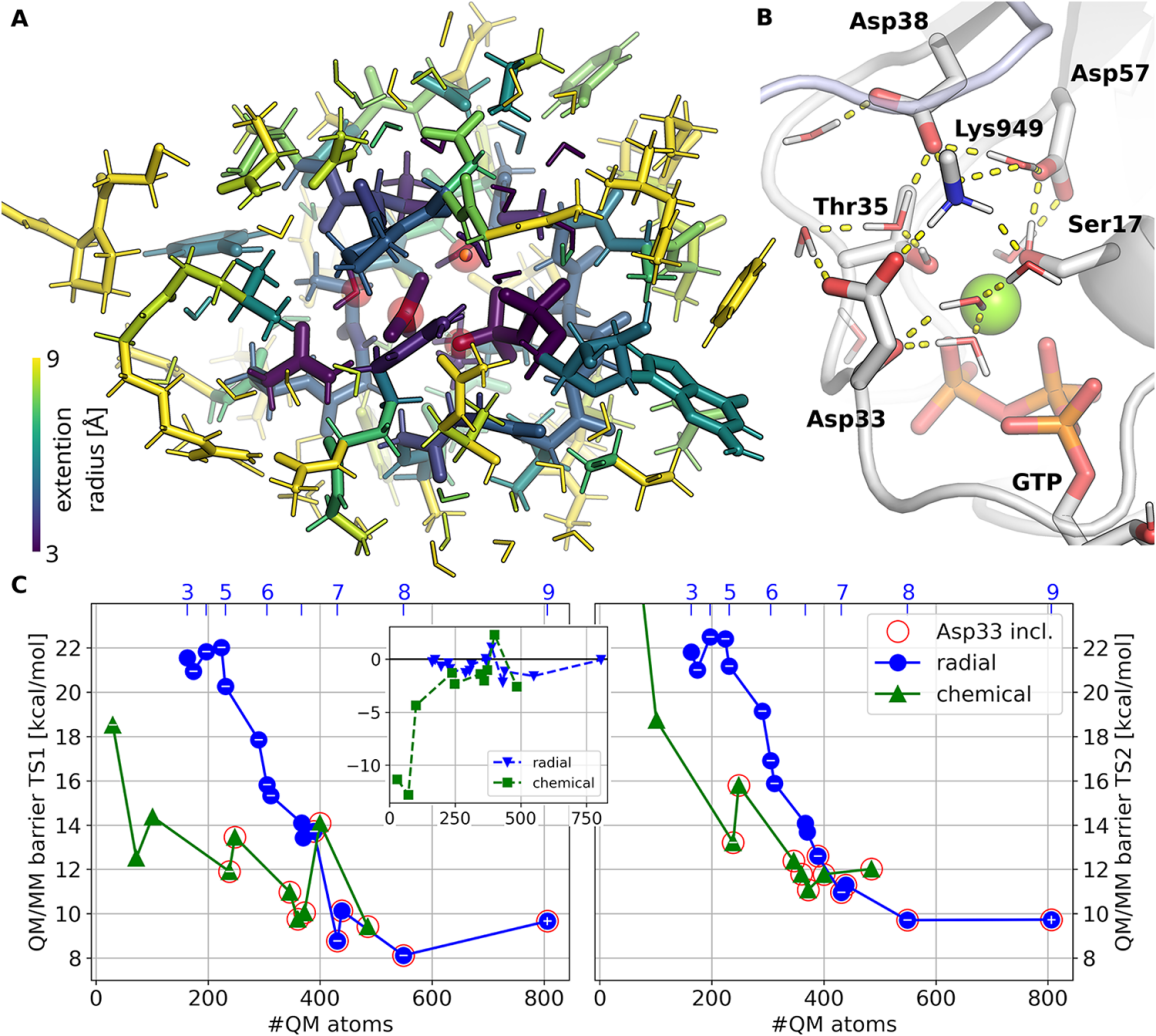
(A) Series of QM selections (30–806 QM atoms, depicted in purple-yellow color scale) extended from four central atoms (Mg^2+^, γ-phosphorus, the oxygen of the nucleophilic water, and the β – γ bringing oxygen) highlighted in red. (B) Hydrogen-bonding network near the Mg^2+^ binding site. (C) Convergence of the QM/MM energies (B3LYP-D3/6–31+G*/CHARMM36m) along two series of QM selections: blue circles: “radial” extension from the reaction center; green triangles: selections prioritizing explicit charges, denoted as “chemical.” Selection radii are depicted for the radial series in the secondary *x*-axis. Formal charge is indicated on the markers if not neutral. Convergence is represented by the two barriers (TS1–RS, left and TS2–RS, right) and their relative energies (TS1–TS2, inset). Selections including Asp33 (and the complete network shown in B) are highlighted by red circles. Other relative energies are presented in Figure S1.

To optimize the QM region, we examined the individual exclusion of several residues (or part thereof) at the edges of QM regions. The moieties should be kept in the QM region if the relative energies change significantly upon their exclusion (Figure S4). The removal of explicitly charged residues had the largest effect on the relative energies. Especially important is Asp33, which is part of a dense hydrogen-bonding network around the Mg^2+^ binding site (Figure 3B).^69^ This suggests that the classical, nonpolarized representation from the protein force field does not adequately describe the interactions between and around explicit charges.^70^ Based on the importance of polar contacts observed in the convergence study, one may reasonably suspect that polarizable embedding in the QM/MM would accelerate the convergence shown in Figure 3. However, the associated cost of the extra self-consistent optimization of multipoles in such a setup was found to be significant (7–11 times vs electrostatic embedding).^71^ Considering the favorable scaling of LESS (section 4.1), the inclusion of residues with significant polarizability near the active site in the LL environment is an affordable and universal solution not requiring parametrization. Interestingly, the removal of water molecules at the edge of this hydrogen-bonding network, but far from the reaction site, appears to have little effect (Figure S4). Although this extensive QM region optimization is not necessary for establishing convergence, it is justified by the cumulative gains from downstream calculations, which is one of the aims of the LASI approach.

Crucially, these selections allowed for the fragmentation of residues at apolar C–C bonds. Let us note that the combination of radial extension with the selection of complete residues only, often used in automated selection algorithms, on average, would constitute a 45% increase in the size of the QM region (Figure S5). In turn, this larger QM size would lead to ca. 5-fold increase in the computational cost at the hybrid DFT level. We also studied whether the number of covalent cuts made at the interface (and the addition of the corresponding link atoms) has any implications for the convergence. We found that neither the number nor the ratio of link atoms (vs the full QM size) alone shows significant correlation with the size-related error. Together with the illustrated importance of the polar networks, this suggests that the quality of the (covalent) cuts at the interface and the insufficiency of the MM model for key polar groups are more important than the size of the QM/MM interface.^72^

Based on these observations, we created selections with minimal extension radii (2–3 Å) around the explicitly charged and key reactive atoms (see Section S1 in the SI). The new series exhibits faster convergence to the asymptotically large QM regions (green series in Figure 3C). We select two QM regions with 238 and 372 atoms that are considerably closer to convergence than the general trends. These are the selections for building the DFT_S_ and DFT_E_ models, respectively, because good convergence in the difference of barriers is reached at 238 atoms, while the same for all relative energies is achieved with 372 atoms. Let us highlight that these selections are optimized for accuracy and would probably be beyond the affordable cost without further acceleration in Section 3.3.

### 3.2 Selecting a QM Method against LNO-CCSD­(T) References

To establish the QM references, the convergence with local approximations and basis sets was assessed in LNO-CCSD­(T)/CBS calculations for three representative QM regions (101, 238, and 372 QM atoms). The basis set dependence exhibits typical convergence patterns (Figures 4 and S6–S7), which are accelerated by the application of the DBBSC. Basis set corrected values are close to chemical accuracy (1 kcal/mol) already with triple-ζ basis sets (triangles in Figure 4), both with and without diffuse functions. The CBS­(T,Q) references are also in agreement with both the cc-pV*X*Z and aug-cc-pV*X*Z series for the 101 (Figure 4A) and 238 (Figure 4B) QM atom cases. Consequently, we omitted the most expensive calculations for the 372-atom model with augmented basis sets. Considering the feasibility and accuracy of the CBS­(T,Q) calculation with DBBSC, we recommend it as a widely applicable reference method for similar (bio)­chemical applications. Namely, LNO-CCSD­(T)+DBBSC for the 101 QM atoms requires ca. 100 and 200 core hours for CBS­(D,T) and CBS­(T,Q), respectively (Figure 4A). The cost increase is about 10-fold for the 238 QM atoms, but still only represents a few days using 16 cores. Then, the 372-atom LNO-CCSD­(T) calculations took about 2000 core hours, indicating that the computations approach the linear-scaling size range. Importantly, the QM size dependence for the LNO-CCSD­(T)/CBS calculations is similar to that of the DFT relative values, which opens up the possibility to calculate the *Δ*Δ*E*_CC_ correction at a reduced cost. This concept is thoroughly discussed in Section 4.2.

**Figure 4:**
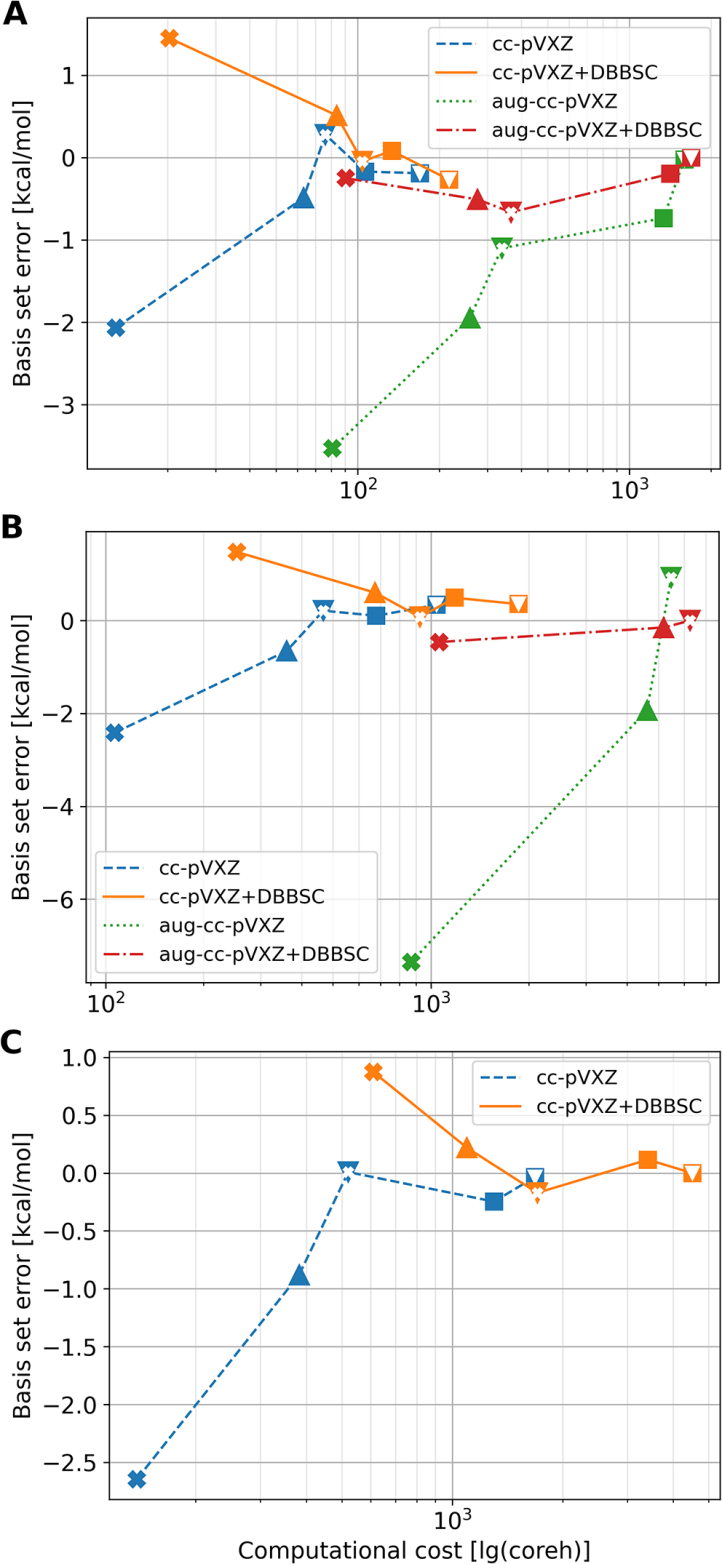
Convergence of the LNO-CCSD­(T) first barrier (**TS1**–**RS**) with basis sets for three QM regions: (A) 101, (B) 238, and (C) 372 atoms, as a function of the computational cost in core hours. Crosses, triangles, and squares refer to cardinal numbers 2, 3, and 4 of the basis sets, combined to depict CBS extrapolation. The basis set errors are measured compared to the best converged CBS­(T,Q) reference. DBBSC stands for the density-based basis set correction. Runtime for CBS data points is the sum of the corresponding two calculations. Basis set convergence for other stationary points is depicted in Figure S6.

The convergence with local-correlation thresholds is rapid, indicated by the LAF uncertainty measures^7,38^ of the local approximation, defined as ±0.5 (*E*_Tight_^*X*^ – *E*_Normal_^*X*^). For the first barrier, this uncertainty is ± 0.14 kcal/mol both with the 101 and 238 QM atom regions with cc-pVTZ, which decreases to ± 0.04 with aug-cc-pVTZ. The highest uncertainty is obtained for the 101-atom cc-pVTZ calculation of the **TS2**, still only ± 0.22 kcal/mol, see more details in Table S1.

To continue with finding DFT models for DFT_E_ and DFT_S_, following our DFT error analysis protocol,^8^ we screened 19 generally well-performing, popular functionals (a few from each rung from GGA to RSH, and the revDSD-PBEP86 double-hybrid) on the 101-atom QM region against LNO-CCSD­(T)/CBS­(aT,aQ) references (Figure 5A). These calculations were performed with the cc-pVTZ basis set and dispersion correction. The detailed numerical results are collected in Table S2 of the SI. Semilocal functionals have a significant overstabilization of the S_N_2-type phosphate cleavage transition state (TS1). Among the funtionals of the most affordable GGA rung, PBE-D3 and PW91-D3 perform the best, but some of their errors are still 5–6 kcal/mol. The performances of hybrid and RSH functionals are similar to each other for all stationary points. Considering the errors on the difference between the two barriers, as a proxy for the quality of describing the chemistry, one of the best performing functionals is PBE0-D3, (0.73 vs the 1.66 kcal/mol of the LNO-CCSD­(T)/CBS references in Table S2). Besides the DFT energies, we analyzed the density sensitivity of functional, in which PBE0, M06–2X, and BHLYP do well among the hybrid rung (see Table S3 and details in section S4.1 of the SI).^8^

**Figure 5:**
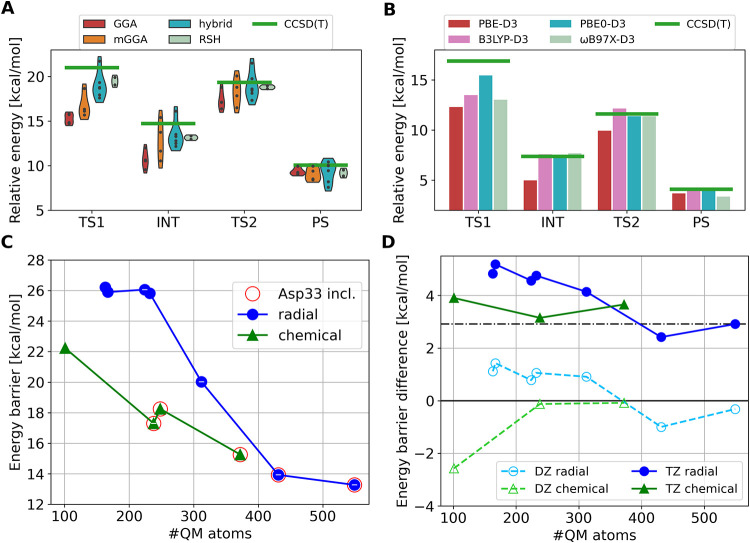
(A) Violin plot of DFT functional rungs compared to the LNO-CCSD­(T)/CBS reference relative QM/MM energies, calculated for the 101-atom QM region. (B) Relative QM/MM energies calculated with selected DFT functionals vs the LNO-CCSD­(T)/CBS references for the 372-atom QM selection. (C) Convergence of the QM/MM energies (PBE0-D3/def2-TZVP/CHARMM36m) along two series of QM selections: blue circles: ‘radial’ extension from the reaction center, green triangles: selections prioritizing explicit charges, denoted as “chemical.” Selections including Asp33 (and the complete network shown in [Fig fig3] **B**) are highlighted by red circles. Other relative energies are presented in Figure S8. (D) Difference in the barrier heights (TS1–TS2) calculated with PBE0-D3 and def2-SVP (DZ, dashed) and def2-TZVP (TZ, solid) basis sets.

Then, for the QM regions where relative energies approach convergence, we also tested a few representative functionals (372 QM atoms, Figure 5B) to study the QM size dependence of the DFT performance. The size dependence of the DFT and reference calculations is similar (Table S4), as is the functional performance for all studied QM sizes. The above test suggests the choice of PBE0-D3 for this specific reaction.

Next, we examined the convergence with the basis sets used at the PBE0-D3 level. Using a double-ζ (DZ) basis is a simple way to limit the computational cost and may be reliable in calculating relative energies if the error is consistent along the configurations. Scanning a series of basis sets up to quadrupole-ζ (QZ), we found that the barrier to **TS1** was underestimated with smaller basis sets, even when diffuse functions are added. Conversely, triple-ζ (TZ) and QZ sets show quick convergence, and def2-TZVP is proven to be a sufficiently large set (cf. def2-SVP and def2-TZVP in Table S5). The DFT basis set convergence trends are consistent across all QM sizes studied (**TS1** barriers of ca. 19.3, 17.4, and 15.3 kcal/mol for 101, 238, and 372 atoms in Table S5, respectively). Most importantly, due to the inconsistent basis set error between the chemical steps, TZ accuracy is the minimum requirement for a qualitatively accurate description of the reaction (Figure 5D). The presented convergence tests demonstrate that the PBE0-D3/def2-TZVP on a 372-atom QM region represents a great choice for DFT_E_. Namely, this level is reasonably converged both in terms of accuracy (against the LNO-CCSD­(T)/CBS references) and in QM size, close to the largest QM regions practically feasible (600–800 atoms).

Upon revisiting the convergence vs the size of the QM region at the DFT_E_ level (Figure 5C), results confirm the convergence at the 372-atom QM selection (rightmost green triangle in Figure 5C,D). The trends of the preliminary QM size screening are the same; selections that prioritize explicitly charged residues over radial extension are converging to the results with large QM sizes faster. Consequently, for the proposed computational scheme in eq 1, this screening identifies the functional (PBE0-D3), basis (def2-TZVP), and QM region size (372 atoms) for the effective DFT_E_ method to approach the LNO-CCSD­(T)/CBS values within about 1.5 kcal/mol (Table S4). This remaining deviation is very well corrected via the ΔΔ*E*_CC_ term of eq 1, see section 4.2.

As we set these parameters, we did not consider the possibility of running extensive free energy calculations beyond the fact that DFT_E_ gradients are readily available. When this DFT_E_ model is too expensive for extensive sampling, one can look for compromises by defining DFT_S_ for sampling extensively. The effect of small basis sets on the two transition states in this two-step reaction is quite unbalanced (DZ vs TZ, 3–4 kcal/mol, Figure 5D and Table S5). On the other hand, shrinking the QM region from 372 to 238 atoms changes the overall barrier from 16.9 kcal/mol to 18.7 at the LNO-CCSD­(T)/CBS level, while the DFT error and the relative position of the two TSs are virtually the same. Therefore, it is more acceptable to make concessions in the size of the QM region than in the size of the basis set. This is particularly important, as a smaller basis set is often employed for optimization even if the final electronic energy is then corrected. With that in mind, the explicit-charge-specific selection of 238 QM atoms at the same PBE0-D3/def2-TZVP level is a good candidate for DFT_S_.

### 3.3 Three-Layer QM_HL_-in-QM_LL_/MM Models

As the QM atom lists and DFT parameters are now identified for DFT_E_ and DFT_S_, we seek to retain their accuracy while accelerating their evaluation. Therefore, we introduce a third layer between the hybrid DFT and the MM regions. As identified above, the high-level, inner QM layer (QM_HL_) is still treated at the PBE0-D3/def2-TZVP level, embedded into a more cost-effective QM_LL_ environment layer. In this section, we elaborate on the selection of the active atom list for the embedded QM subsystem and the level of theory for the outer QM_LL_ layer, corresponding to the total QM_HL_+QM_LL_ region of 238 atoms.

We start with the Huzinaga-embedding and the selection of the QM_LL_ model. Similar to the good performance of PBE0-D3 among hybrid functionals, PBE-D3 worked well at the GGA rung; therefore, we selected it for QM_LL_ and screened the active atom selections. In general, it is good practice to select similar functionals for QM_HL_ and QM_LL_.^52^ As discussed in Section 2.3, in our quantum embedding implementation, the starting point of the HL SCF calculation is created from the LL orbitals of the whole system. Consequently, the selection of the active subsystem in the Huzinaga embedding is more flexible, and it does not require link atom definitions. Conveniently, formal charges are not needed to be individually assigned to all QM subsystems, like in ONIOM methods. An additional key difference compared to QM region selection (in QM/MM) is that the Huzinaga embedding interface can split polar bonds, even those with the bond order above one, or moderately delocalized moieties.^73^ This enables smaller subsystems to be chosen for the more expensive QM_HL_ calculations. Initially, besides the reactive moieties (the triphosphate anhydride, the glutamine (Q61), and the nucleophilic water), we added all of their counterions to the QM_HL_ atom list, namely a lysine (K16), an arginine (R789) and the Mg^2+^ with all of its coordinating ligands. This selection could be further shrunk by removing the lysine and arginine as well as the α-phosphate, arriving to 28 active atoms and 87 occupied MOs (Figure 6A). Interestingly, for these two elementary reaction steps, removing the Mg^2+^ ion with the alcohols and waters in its coordination sphere still works well (see 17 QM_HL_ atoms in Figure 6C). We anticipate this partitioning would break down if the Mg^2+^-phosphate contact is broken, so for greater universality, we consider only the 28 active atom selection hereafter. Overall, the quantum-enhanced calculations reliably produce results within 0.5 kcal/mol of the complete QM_HL_ relative energies. This holds as long as all the reactive moieties are treated at the QM_HL_ level, which we also found in other types of reactions.^35^ This consideration ensures that the moieties described with large semilocal DFT errors are treated at the hybrid QM_HL_ level, such as the S_N_2 center of the phosphate cleaving **TS1**.

**Figure 6:**
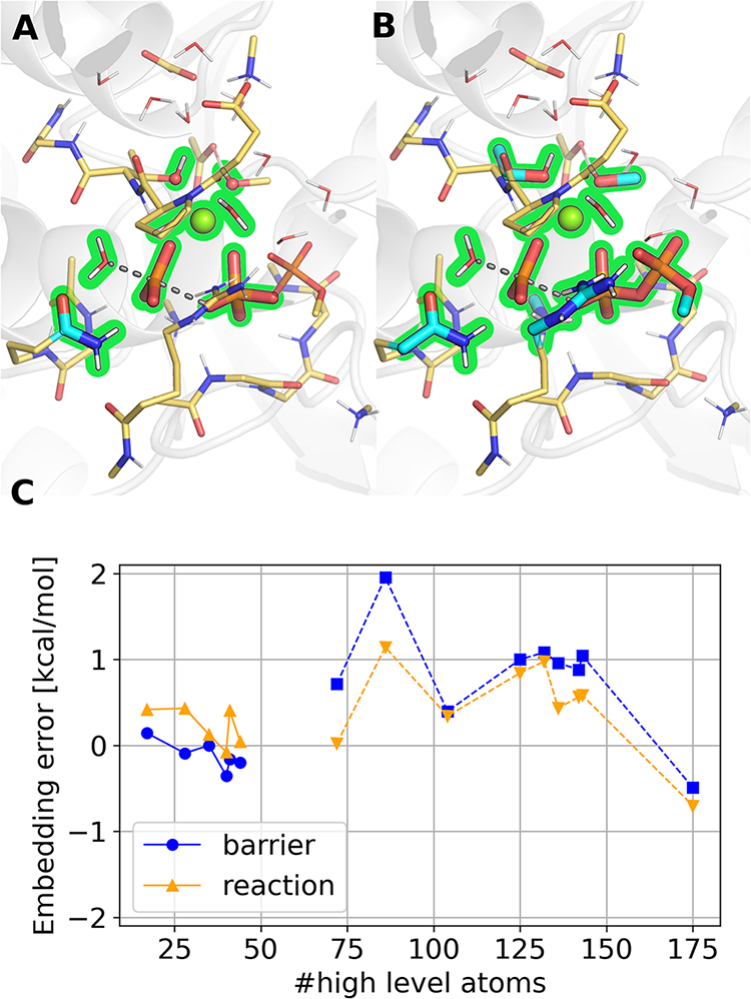
Active subset selection for quantum (A = 28 atoms) and ONIOM embeddings (B = 72 atoms). (C) Errors in relative energies against pure high-level (PBE0-D3/def2-TZVP) calculations with different active atom lists in Huzinaga (solid lines) and ONIOM embedding (dashed lines). The environment in both cases was described at the PBE-D3/def2-SVP level of theory. Errors depicted for the first (overall) barrier (TS1-RS) are colored blue (circles for Huzinaga quantum embedding, squares for ONIOM), and for the reaction energy (PS-RS), yellow (upward triangles for Huzinaga quantum embedding, downward triangles for ONIOM).

For an established 28-in-238 QM_HL_-QM_LL_ split of the total QM region, we tested shrinking the AO basis set for the environment method. Despite the pronounced basis set error depicted in Figure 5D, this mixed-basis approach (QM_HL_: PBE0-D3/def2-TZVP in QM_LL_: PBE-D3/def2-SVP) introduced negligible errors (<0.4 kcal/mol, see Table S6) in the relative QM_HL_-in-QM_LL_/MM energies. The errors of these quantum embedded, mixed-basis calculations (vs the whole system treated at QM_HL_ level) are depicted in Figure 6C for active regions between 17 and 44 atoms. Employing the LESS framework to further accelerate the Huzinaga-embedded calculations introduces negligible deviations (0.2–0.3 kcal/mol); we discuss this further focusing on the achieved acceleration in Section 4.1. For general cases, uncertainties related to the basis set truncation introduced in LESS are tightly and systematically controllable by the associated thresholds.^35^

To compare the performance of the Huzinaga quantum embedding to the most commonly used multilayer scheme, ONIOM, we created a different series of subdivisions of the total QM region. Importantly, the selection rules for the active atoms have to be similar to those used at the QM/MM interface for keeping chemical integrity and for the definition of link atoms (hydrogens) in the HL ONIOM subsystem. This causes practical limitations as opposed to the flexible and effective region splitting enabled by the Huzinaga quantum embedding. Furthermore, unlike the Huzinaga embedding, the HL ONIOM calculations require the definition of charges for the LL atoms. Besides using the MM charges, they can be defined in multiple ways, for example, based on the LL QM calculations, as explored in Section S5.2 in the SI. As a direct comparison to the quantum embedding, HL: PBE0-D3/def2-TZVP in LL: PBE-D3/def2-SVP ONIOM calculations were benchmarked with active atom lists of between 72 and 175 atoms (Figure 6B). It is cautionary that the ONIOM relative energies do not show convergence below 100 HL atoms, and the ONIOM errors are twice as high as those of the Huzinaga quantum embedding, even when 75% of the total QM region (175 out of 238 atoms) is in the ONIOM HL layer (Figure 6C and Table S7). One advantage of the simple subtractive scheme of ONIOM is that an even more cost-effective LL method can be used for the environment. For example, semiempirical QM methods can be considered, such as tight-binding DFT, which are not compatible with the Huzinaga embedding. We tested GFN2-xTB as an affordable tight-binding method for the LL QM region, but the relative energies were insufficient for our ambitious accuracy purposes (Figure S9).

## 4 Discussion and General Suggestions

### 4.1 Accuracy as a Function of Computational Cost

We established that both DFT_E_ and DFT_S_ require the accuracy of the hybrid/TZ level of DFT, while the difference between them is the size of the QM regions. We also demonstrated that this accuracy can be maintained with the application of a mixed-basis set and Huzinaga quantum embedding. The relative energies converge after 372 QM atoms, while at 238 atoms, the difference of the barrier heights provides reliable chemical conclusions. Now turning to the affordability aspect, A3, Table 1 and Figure 7 summarize the computational cost of methods for both the 238- and 372-atom QM regions. The QM_HL_ method, PBE0-D3/def2-TZVP, is selected as the reference for the error in the barrier height, and speedup factors are calculated against that same reference for both QM region sizes. For comparison, PBE-D3/def2-SVP results are also depicted (top right in Figure 7), which have a large error combined from the functional and the basis set (−8.86 and −7.72 kcal/mol) but are faster than PBE0-D3/def2-TZVP by 2–3 orders of magnitude.

**Figure 7:**
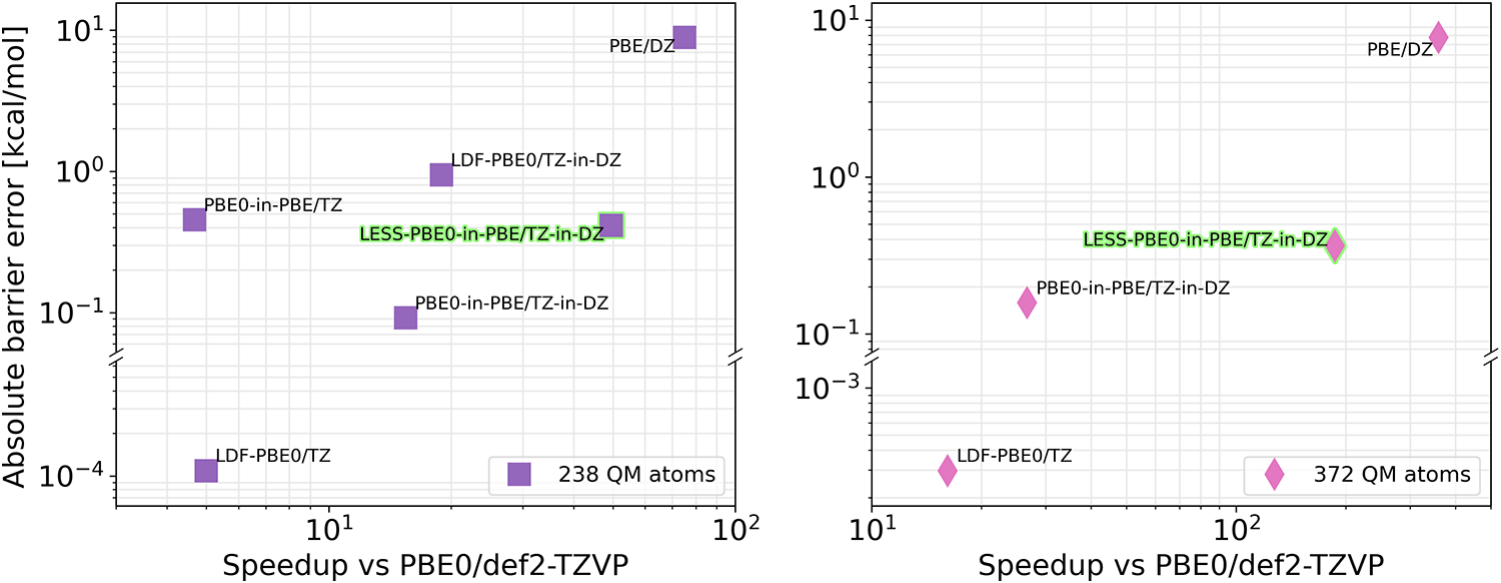
Absolute barrier error vs speedup achieved against the approximation-free PBE0-D3/def2-TZVP calculations in kcal/mol for the 238 (left) and 372 (right) QM atom selections. DZ and TZ stand for def2-SVP and def2-TZVP basis sets, the -D3 labels are omitted for clarity.

**Table 1:** CPU Time Requirement and Accuracy of DFT and DFT-in-DFT Calculations

method^a^	basis	approx.	QM atoms	CPU^b^ time [coreh]	barrier error [kcal/mol]	speedup
**PBE0**	**def2-TZVP**	**reference**	**238**	**138.7**		**1.0**
PBE	def2-SVP		238	1.8	–8.86	75.0
PBE0	def2-TZVP	LDF	238	27.8	0.00	5.0
PBE0	TZ-in-DZ	LDF	238	7.3	–0.94	18.9
PBE0-in-PBE	def2-TZVP		28-in-238	29.6	0.45	4.7
PBE0-in-PBE	TZ-in-DZ		28-in-238	9.0	–0.09	15.5
PBE0-in-PBE	TZ-in-DZ	LESS	28-in-238	2.8	–0.41	49.5
						
**PBE0**	**def2-TZVP**	**reference**	**372**	**1045.1**		**1.0**
PBE	def2-SVP		372	3.7	–7.72	281.6
PBE0	def2-TZVP	LDF	372	82.6	0.00	12.7
PBE0-in-PBE	TZ-in-DZ		28-in-372	50.1	0.16	20.9
PBE0-in-PBE	TZ-in-DZ	LESS	28-in-372	7.1	0.37	146.2

a All results include D3 empirical dispersion correction.

b Ran on 16 cores of an Intel Xeon Gold 6448H CPU without hyperthreading.

Starting with the LDF-accelerated hybrid results, they agree with the conventional PBE0-D3 results within 10^–3^ kcal/mol, while being 5–12 times faster. Moreover, the speedup naturally increases with the system size (bottom left in Figure 7).

We tested a few additional combinations of approximations for the 238-atom QM regions. Our Huzinaga quantum embedding implementation without further approximations also fares well for the barrier, and it introduces a similar speedup to that of LDF (5.0 for LDF and 4.7 for Huzinaga). Introducing a smaller basis set to the QM_LL_ method (TZ-in-DZ mixed basis) results in a 3-fold increase in speed for quantum-embedded calculations (from 4.7 to 15.5) without a penalty in accuracy. A similar mixed-basis approach combined with LDF, but without embedding, also comes with a similar speedup (from 5.0 to 18.9), although with a 0.94 kcal/mol error on the reaction barrier measured. The Huzinaga-embedded calculations with mixed-basis (−0.09 kcal/mol error, 15.5× speedup) come at a similar cost to the LDF-PBE0/mixed-basis runs, but with the accuracy closer to that of the slower PBE0-in-PBE/TZ or LDF-PBE0/TZ methods.

The ultimate acceleration is, however, achieved by our LESS framework, which scales well with the system size, providing 50-fold and 146-fold speedups for the 238-atom and 372-atom QM regions, respectively. In these runs, the GGA-level QM_LL_ environmental SCF computation takes up more than half of the total execution time (cf. the PBE/def2-SVP speedups of 75 and 282 over PBE0/def2-TZVP). The LESS scheme, beyond the speedup of the approximation-free Huzinaga embedding, benefits from the effectiveness of the mixed-basis approach and the truncation of the AO and DF basis sets in the QM_HL_ calculations. Furthermore, its efficiency is further enhanced by its compatibility with the in-core integral storage for the QM_HL_ calculation due to the LESS approximations.^35^ Overall, the LESS QM_HL_-in-QM_LL_ framework offers close to QM_HL_ accuracy for the cost of about two QM_LL_ calculations. In practical terms, if LESS is employed in the DFT_S_ model, a single-point energy calculation takes 10 min wall time on 16 cores at an Intel Xeon Gold 6448H CPU (2.8 core hours). This warrants the processing of tens of thousands of configurations within reasonable simulation times. DFT_E_, which in this study differs only in the size of the QM region, would require about 3-times more resources per geometry, which makes exhaustive PES exploration sufficiently affordable.

The affordability of the herein-reported QM models also makes them good candidates for structure and path reoptimization, according to our preliminary calculations. These will be thoroughly examined once the gradient calculations for LESS are available. All in all, the combination of DFT_S_ efficiency and CCSD­(T)-corrected DFT_E_ energetics forms a very good balance between the three aspects considered here that are accuracy (A1), QM size (A2), and cost (A3).

### 4.2 Efficient Enthalpic Correction via LNO-CCSD­(T)

The LNO-CCSD­(T) correction is used alongside DFT_E_ for a discretely represented reaction path. Here, we used it for a few stationary points and also characterized its cost to show its applicability for a larger number of conformers. The broader affordability enables the consideration of the LNO-CCSD­(T) correction to improve the quality of the free energy profile, e.g., via thermodynamic perturbation.^74,75^ It is worth noting that recent applications related to ML potentials highlight the difficulties in connecting ensembles from different levels of theory, while also providing well-founded ways to overcome them.^76,77^ While one may expect that the DFT_E_ or DFT_S_ methods selected to best reproduce the highly accurate references for the specific application provide sufficiently close ensembles, this assumption requires future validation.

For the largest, 372-atom QM regions, we carried out LNO-CCSD­(T)/CBS calculations showing that convergence within chemical accuracy requires CBS­(D,T) basis extrapolation, taking more than 1000 core hours, while CBS­(T,Q) results with DBBSC cost more than 4000 core hours. Figure 8 depicts that the well-performing DFT_E_ method has an uneven error along stationary points with respect to the LNO-CCSD­(T)/CBS reference (blue dashes). When smaller QM regions are examined, the size-related error of the LNO-CCSD­(T)/CBS and DFT_E_ calculations is similar (orange dots and green dot-dashes, respectively). In other words, the LNO-CCSD­(T) correction term Δ*E*_CC_, defined in eq 1 converges faster with the QM size than the relative energies and thus can be calculated at smaller QM sizes. In these CC-corrected composite references of DFT_E_ + Δ*E*_CC_, the relative barrier heights are at the quality of the LNO-CCSD­(T)/CBS calculations, and the average error of the relative energies is 0.2–0.4 kcal/mol (Table 2). Crucially, the computational cost of these smaller LNO-CCSD­(T)/CBS calculations with the 101-atom Δ*E*_CC,101_ correction is only 5% of that of the large, 372-atom model, ultimately allowing chemical accuracy to be achieved for 20-times more configurations. To put this into perspective, the 217 core hour demand of the Δ*E*_CC,101_ calculations is on the same order as a hybrid DFT calculation of the 238-atom QM model without approximations.

**Figure 8:**
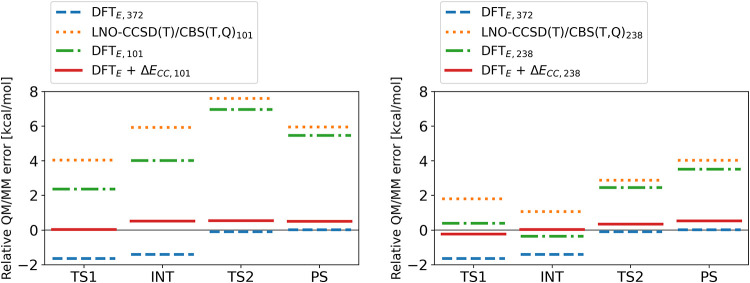
Errors in the relative QM/MM energies of stationary points using 101 (left) and 238 (right) QM atoms for the Δ*E*_CC_ correction evaluated against the largest (372-atom) LNO-CCSD­(T)/CBS reference values.

**Table 2:** Relative Barrier Heights, Mean Absolute Errors (kcal/mol), and Indicative Computational Cost [coreh] for LNO-CCSD­(T)/CBS, DFT Calculations, and Composite Schemes

method	QM atoms	Δ*E*(**TS1**–**TS2**) [kcal/mol]	MAE [kcal/mol]	CPU time [coreh]
LNO-CCSD(T)/CBS(T,Q)	372	5.21		4508
LNO-CCSD(T)/CBS(T,Q)	238	4.13	2.45	1856
LNO-CCSD(T)/CBS(T,Q)	101	1.66	5.88	217
DFT_E_: LESS-PBE0-in-PBE/TZ-in-DZ	372	3.65	0.79	7.1
DFT_S_: LESS-PBE0-in-PBE/TZ-in-DZ	238	3.15	1.68	2.8
LDF-PBE0-D3/def2-TZVP	101	0.62	4.70	1.0
DFT_E,372_ + Δ*E*_CC,238_		4.64	0.17	1866
DFT_E,372_ + Δ*E*_CC,101_		4.70	0.40	225

### 4.3 General Advice on Model Building

The gain from the appropriate application of QM_HL_-in-QM_LL_/MM with negligible compromise in the high-level method’s accuracy provides an opportunity to elevate the overall enzyme simulation practice. However, the introduction of the interfaces at QM/MM and QM_HL_-QM_LL_ boundaries, selection of methods, and atom lists for each layer add some complexity to the model definition. Therefore, we compile the conclusions from our experience into transferable advice for setting up simulations for enzymatic reactions based on the introduced systematically converging strategies. Starting from an atomistic model (typically from experimental structures), we suggest the following pipeline:

1. Choose a reasonable, moderate-sized QM region and DFT functional for preliminary calculations.
2. Obtain a preliminary reaction path (at least reactant, product, and a few plausible intermediates and TSs) to represent the chemical space.
3. Establish the convergence of relative energies with the size of the QM region (A2 optimization, as proposed in Section 2.3).
4. Select an appropriate DFT functional and basis set against reliable references (QM accuracy (A1) optimization).
  (a) Select an accurate high-level (HL) DFT method and basis set for converged energetics [e.g., wrt. LNO-CCSD­(T)].
  (b) If warranted by QM region size, select an efficient low-level (LL) DFT method and basis set for quantum embedding.
  (c) Confirm size-convergence with the selected QM_HL_ approach. (A1+A2 optimization)
5. Optimize HL active atom list of the QM_HL_-in-QM_LL_ embedding reproducing the QM_HL_ results.
  (a) If aiming also at sampling, select DFT_S_ e.g., further trim the total QM region if necessary. (A1+A2+A3 optimization)
6. Optimize/explore reaction paths (DFT_E_) and proceed to free energy simulations (DFT_S_).
  (a) If substantially different mechanisms are discovered, revisit steps 3–4.
7. Sample along key paths of *step 6* again and apply affordable Δ*E*_CC_ corrections via LNO-CCSD­(T).

From a starting structure, typically representing the reactant or the product state of the reaction, one can define QM regions and start *step 3* of converging reaction energies. However, we recommend the preliminary exploration of the chemical reaction(s) for two main reasons. First, it is important to include nonequilibrium geometries in the set of structures in which the convergence of the QM/MM model is tested. The overall reaction and thus which bonds are breaking and forming are known, so a preliminary reaction channel can be created easily. The intermediate structures identified this way are expected to be more representative of the DFT uncertainties specific to the overall reaction than the reactant and product states alone (such as the S_N_2 TS in this example). Therefore, including them enables more robust QM method selection for the bond rearrangements, even if alternative paths are considered later. Second, depending on the reaction, the involvement and proximity of residues may change along the reaction coordinate, potentially resulting in an unbalanced representation in the QM region along the reaction coordinate when only the reactant is considered. This way, important interactions along the path (like polar contacts presented here) can be better understood when determining the size of the QM region that is used later for the mechanistic exploration. In the current work, the testing structures for *steps 1–2* were taken from a previous study.^20^

The general conclusion of *step 3* is that several hundred QM atoms may be required for converged properties. Our case study shows that the convergence is not necessarily monotonic; therefore, accurate reference calculations and methods providing systematic convergence are extremely helpful for robust model selection. It is reasonable to assume that in the general case, a purely radial selection is suboptimal. Instead, following physical principles, such as prioritizing representation of explicit charges and polar contacts, is expected to result in more effective QM region definition. Importantly, selection algorithms are more efficient if they use fragments smaller than residues as the nondivisible unit, since most amino acid residues can be split along various apolar bonds at the QM/MM interface.

For the accuracy of the electronic structure method itself, gold-standard local-correlation methods, especially the highly optimized LNO-CCSD­(T)^38^ variant, are available for even the presumed several-hundred-atom QM region sizes, thus benchmarking production DFT functionals is clearly feasible (*step 4*). Studies indicate the need for hybrid or higher rungs of functionals, especially for accurate activation energies when it is helpful to suppress DFT errors, such as polarization and overdelocalization. When optimizing for speed, one might compromise on the size of the basis set, but caution is advised, especially when different chemical steps are compared. Uneven error sources may change the conclusions qualitatively, as demonstrated on the basis set dependence of the relative barrier heights here. From the DFT benchmark, one can also gain information for an efficient QM_LL_ method used for the QM environment. Once the optimal functional-basis combination is found, the size dependence studied in step 3 should be revisited.

Having the two-layer QM/MM baseline, in *step 5*, the split of the QM regions by Huzinaga-based embedding can be intuitively constructed with a small active atom list (e.g., the reactive atoms) without strict rules on the fragmentation. The interface does not require a special definition; the only pivotal point is to make sure that the orbital selection is consistent along the reaction path. Robust orbital selection algorithms ensure this for sufficiently large QM_HL_ sizes beyond the region affected by the bond forming and breaking (although highly delocalized or metallic materials may be sensitive to this issue).^49^ Besides the speedup quantum embedding offers, further techniques such as a mixed-basis definition for the QM subsystems, and ultimately the basis set truncation of our new LESS framework^35^ can be greatly exploited. The individual approximations each constitute a 2–10-fold acceleration with well-controlled errors on the relative energies, making LESS QM_HL_-in-QM_LL_ embedding up to 2 orders of magnitude faster than the conventionally required hybrid DFT methodology. In other words, in *step 5*, one establishes the DFT_E_ component of LASI, while in *step 5a* defines DFT_S_. Concessions on the size of the QM region are justified if sampling methods necessitate them, especially if the chemical conclusion remains intact and the introduced errors are well controlled by the underlying convergence study. In the presented case, the bespoke 238-atom region is deemed a reasonable compromise that can be used in subsequent free energy simulations, while the energetic term can be obtained with a better converged DFT_E_ approach.

*Step 6* is where the (bio)­chemical exploration takes place. Probing different mechanistic alternatives or free energy simulations can be executed with the selected LASI component in an affordable and accurate fashion. If unforeseen reaction steps or intermediates are found with the more accurate model, in *step 6a*, one should verify the convergence and accuracy of the QM/MM model selected in *steps 3–5* on the new structures and path obtained with the optimized model. In other words, one should note the possibility that the initial model used to generate the initial structures and path in *steps 1–2* may substantially differ from the optimized model. In such cases, *step 6a* recommends revisiting *steps 3–5* on structures and path refined with the optimized method. The potential necessity and impact of this step are under investigation for the studied RAS reaction, which will be reported in our forthcoming work. All in all, such extended mechanistic studies are enabled by two crucial criteria in our model selection: the feasibility of gradient calculations for the QM­(-in-QM) model, enabling optimization and molecular dynamics, and restricting the calculation cost (i.e., wall time), ensuring practical affordability.

Finally, based on observations made in *step 4*, the application of Δ*E*_CC_ correction to DFT energetics may be very useful or even necessary to reliably approach chemical accuracy. Furthermore, one may expect that due to similar QM size effects at the LNO-CCSD­(T) and (well-selected) DFT levels, the incorporation of the Δ*E*_CC_ correction is sufficient using a smaller QM size, significantly decreasing the computational cost of the correction to the range of hundreds of core hours.

We recognize that not all simulations aim to calculate the free energies. We summarize the options the presented pipeline provides in Table 3. For potential energies, the DFT_E_ model should be effective, and then *step 5a* will not be necessary. If reliable benchmarks are available, one may choose to simplify *step 4* and/or *step 7*, but it is important to emphasize that simulation parameters can be coupled and method (functional) selection should also be informed by QM size dependence. Nevertheless, in chemical applications where the entire system may be described at the QM_HL_ level affordably, *steps 3* and *4c* are obsolete. While the number of demonstrations is limited, the generality of our systematic approach suggests that, a wide variety of applications would benefit from the acceleration that the novel LESS quantum embedding,^35^ DBBSC^78^ and LNO-CCSD­(T)^7^ schemes offer.

**Table 3:** Utility and Performance of the Components of the LASI Thermodynamic Scheme

	DFT_E_	Δ*E*_CC_	DFT_S_
goal	optimization, PES exploration	chemical accuracy	FES exploration
method	LESS hybrid-in-GGA	LNO-CCSD(T)/CBS	LESS hybrid-in-GGA
accuracy	1–1.5 kcal/mol	0.5–1 kcal/mol	<2 kcal/mol
QM size	300–400 atoms	≈100 atoms	≈200 atoms
comp. cost	10 coreh	200 coreh	3 coreh

## 5 Conclusions

This work presents a comprehensive strategy to advance the accuracy and efficiency of QM/MM simulations for enzymatic reactions, focusing on the feasible computational cost while retaining high accuracy. Inspired by the key characteristics of locality accelerated and systematically improvable, the new approach is labeled as LASI. Through systematic convergence studies on the QM region size and benchmarking against high-level local coupled-cluster reference calculations, our results echo that several hundred atoms could be required to achieve chemically accurate representations of enzymatic reaction energetics. Based on the presented strategy, a well-converged DFT_E_ methodology and a computationally lighter DFT_S_ model can be selected. DFT_E_ is ideal for the exploration of the potential energy surface, while DFT_S_ can be employed to efficiently explore reaction paths and perform free energy simulations to obtain entropic contributions. Furthermore, we suggest an LNO-CCSD­(T)-based Δ*E*_CC_ correction to the relative electronic energies and show that its application to DFT_E_ is effective and affordable, resulting in chemically accurate energetics even with 300–400 QM atoms. These levels of the computational scheme enable users to tailor their simulation protocol to available resources while confidently maintaining the best accuracy through systematic convergence and embedding strategies outlined in the study.

To make the DFT_E_ and DFT_S_ methods even more affordable, we employ our recently introduced LESS quantum embedding framework. It combines a Huzinaga-equation-based embedding scheme with basis set truncation, accelerating the embedded hybrid DFT calculations by up to 2 orders of magnitude, while preserving the requisite precision of the high-level calculation. This layered QM_HL_-in-QM_LL_/MM framework significantly surpasses the accuracy and efficiency of traditional ONIOM approaches, especially due to the flexible active atom selection and reduced basis sets in the environment. Importantly, we demonstrate that the method can handle complex enzymatic environments with highly polarized and charged active sites.

With the achieved speed-up, path optimizations and free energy simulations of (bio)­chemical systems become affordable with the sufficiently converged LASI model. The overall aim of LASI simulations will soon be enabled by the development of analytical gradients for the LESS approach. Thus, its application in refining the reaction path sampled here will be the focus of our forthcoming study, which will also reveal whether our optimized model has any dependence on our initial reaction path. Ultimately, we aim to move toward free energy simulations with the present biological system and beyond.

Our case study on Ras GTPase hydrolysis exemplifies the necessity of charge-aware QM region selection, surpassing simple radial extensions, and highlights that the nearby inclusion of polar contact networks is essential for convergence. The proposed workflow from preliminary path sampling through systematic convergence and embedding optimization into free energy simulations provides practical and transferable guidelines for setting up robust enzymatic reaction models in general. Overall, the new LASI framework paves the way for reliable and computationally feasible QM/MM simulations that can meaningfully complement experimental studies and aid in the rational design of biochemical catalysts.

## 6 Computational Details

### 6.1 System and QM/MM Setup

As prepared in ref 20, the finite (nonperiodic) QM/MM system (7815 atoms) was based on the PDB structure 1WQ1 containing the 5092 protein atoms and 906 water molecules environment. Electrostatic embedding and hydrogen link atom scheme were utilized in all calculations at the QM/MM interface, the MM terms for the link atoms were excluded from the energy evaluation. The MM terms were calculated by CHARMM 47b2, with the CHARMM36m and TIP3 force fields.^57,79–81^ The nonbonding interaction energy MM terms were switched off in the range of 11–12 Å. For all calculations, we considered five stationary points identified in the previous works at the B3LYP/6–31+G* level of theory (Figure 1).

### 6.2 LNO-CCSD­(T) Reference Calculations

LNO-CCSD­(T) calculations employed the (aug-)­cc-pV*X*Z basis sets (X = D, T, and Q) on the first and second row elements and (aug-)­cc-pV­(*X*+d)­Z for the phosphorus.^82^ Weighted core–valence (aug-)­cc-pwCV*X*Z^83,84^ basis set was used for the Mg^2+^ ion to account for the subvalence correlation of the 2s and 2p shells. We performed CBS extrapolations on the HF^85^ and correlation^86^ energies [CBS­(*X*,*X*+1)] to further accelerate the basis set convergence. Corrections from the complementary auxiliary basis set (CABS) to the HF, and the recent LNO-^39^ and density-based basis set correction (DBBSC) to the CCSD­(T) correlation energies were added.^78^ LNO settings are used as defined in ref 38, and as implemented in the Mrcc program suite.^40–42^

### 6.3 DFT and DFT-In-DFT Calculations

DFT calculations were carried out with Mrcc^40–42^ with various functionals and the def2-*X*(Z)­VP­(PD) basis sets (X = S,T,Q).^87^ Active MOs in the quantum embedding calculations were selected based on Mulliken population analysis with a threshold of 0.3. In LESS calculations, AO and DF basis set truncation were based on the Mulliken and LDF algorithms, with thresholds 10^–4^ and 2.0, respectively.^35^ In all calculations, electrostatic embedding was used to best describe the environment and avoid issues with Kohn–Sham iterations.

### 6.4 Region Extension Algorithm for QM Region Definition

Our QM selection utility is available in the following repository: https://github.com/bertadenes/qm_selector. We considered all five stationary points when selecting the QM regions. Upon the initial selection of key QM atoms, an extended core region of atoms with a given radius was defined, with hydrogen atoms included. This region was iteratively extended along the covalent bonds defined in the MM topology, and a link atom was initialized when a cutable bond was reached. Aliphatic fragments up to two carbon atoms were excluded if yielded by the selection. The produced inputs were processed by CHARMM to create the QM inputs with correctly placed hydrogen link atoms.

## Supplementary Material







## Notes

Detailed results of convergence with the size of the QM region, basis sets, local-correlation thresholds; results of the functional benchmark and density sensitivity; Uncertainties related to embedding and ONIOM results (PDF)
Atom indices for entire QM and active subsystem selections (XLS)
Classical topology of the nonperiodic MM system (PSF); Cartesian coordinates of the studied stationary points (CRD) (ZIP)
