# Supplementary material for: Systematically Improvable and Locality Accelerated Enzymatic Reactivity Modeling: Toward Chemical Accuracy at Affordable Cost
Source: J Chem Theory Comput. 2026 Mar 24;22(7):3415–30. doi: 10.1021/acs.jctc.5c02128 (PMC13085240; doi:10.1021/acs.jctc.5c02128)
Supplement: Supplementary file 1 [file ct5c02128_si_001.pdf]

Supporting Information for Systematically Improvable and Locality Accelerated Enzymatic Reactivity Modeling: Toward Chemical Accuracy at Affordable Cost

Dénes Berta,[†,‡,¶] József Csóka,[†,‡,¶] Gyula Samu,[†,‡,¶] and Péter R. Nagy[*,†,‡,¶]

†*Department of Physical Chemistry and Materials Science, Faculty of Chemical Technology and Biotechnology, Budapest University of Technology and Economics, Műegyetem rkp. 3., H-1111 Budapest, Hungary*

‡*HUN-REN–BME Quantum Chemistry Research Group, Műegyetem rkp. 3., H-1111 Budapest, Hungary*

¶*MTA–BME Lendület Quantum Chemistry Research Group, Műegyetem rkp. 3., H-1111 Budapest, Hungary*

E-mail: nagy.peter@vbk.bme.hu

S1 QM selections

One-based atom index of the core selection used for explicit charge specific "radial" selections

```
196, 471, 545, 5107
```

One-based atom index of the core selection used for explicit charge specific "chemical" selections

`196, 209, 470, 471, 494, 494, 501, 544, 545, 766, 767, 812, 813, 1708, 2807, 3977, 4637, 5048, 5081, 5082, 5083, 5084, 5085, 5086, 5087, 5088, 5089, 5090, 5091, 5092, 5098, 5104, 5107`

The lists including the 1-based indices of the QM regions discussed in this work can be found in the corresponding `csv` file.

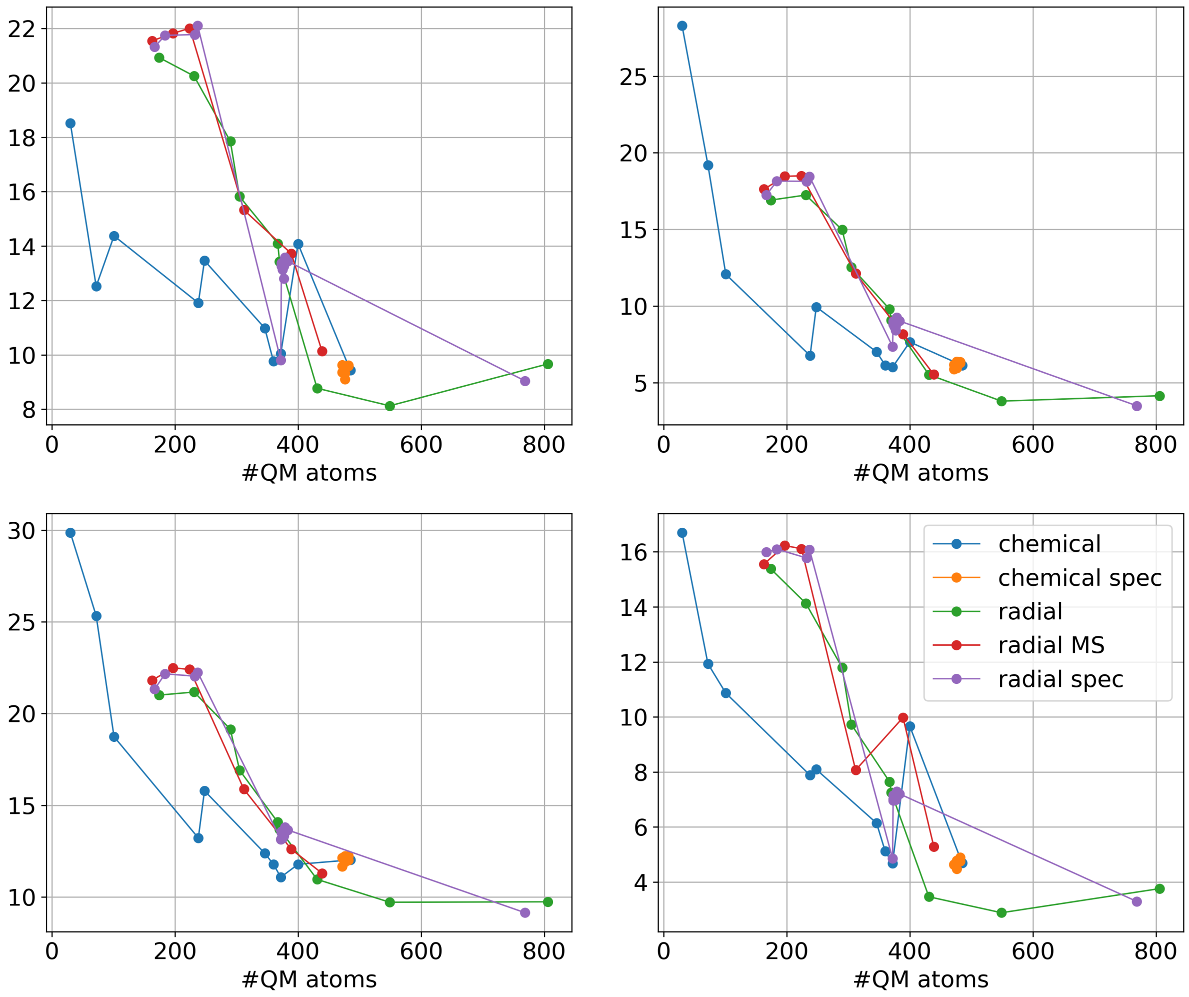

Figure S1: Convergence of the B3LYP-D3/6-31+G*/CHARMM36m relative energies (vs the reactants state RS) of the four other stationary points. **Needs labels: top row TS1, INT, bottom row: TS2, PS** Grouping needs consideration: **spec** means it is a modified version of another selection (e.g. a residue added or removed), **MS** means it was created based on multiple structures. In the main text, **spec** selections are omitted.

S2 Convergence of Specific QM/MM Terms

The relative MM contributions would naturally vanish with an infinite QM size, but their convergence to zero is fairly slow, as depicted in Figure S2. Vanishing MM contributions can be considered as an upper limit of QM size convergence. However, the QM/MM convergence with QM size is faster, as replacing QM with MM contributions to the relative energy is reliable sooner than the converence of cluster models.

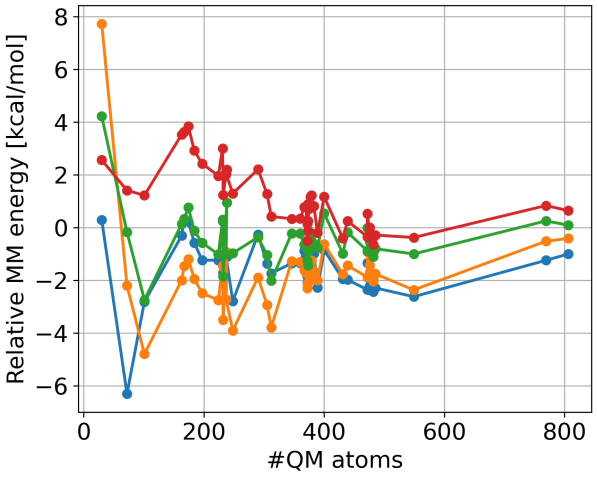

Figure S2: Convergence of the MM part of the relative energies (left) and dispersion correction terms (right) in all selections.

Unlike the MM relative energies, the D3 empirical dispersion correction summed over the QM atoms does not vanish, but tends to the dispersion energy contribution of the investigated reaction energy. It is rather sensitive to the QM selection and thus not a good convergence indicator on its own.

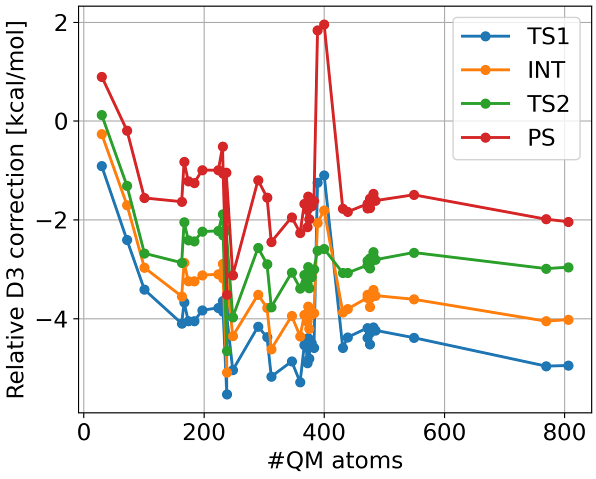

Figure S3: Convergence relative D3 correction energies in all selections, calculated for the B3LYP hybrid functional.

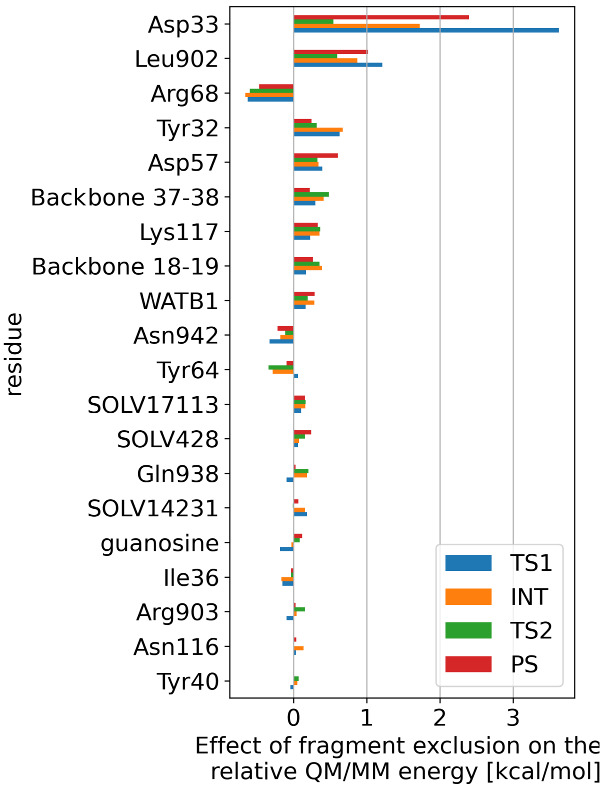

Figure S4: The effect of localized changes by removing/adding a single residue or backbone section in the relative QM/MM energy of stationary points in kcal/mol.

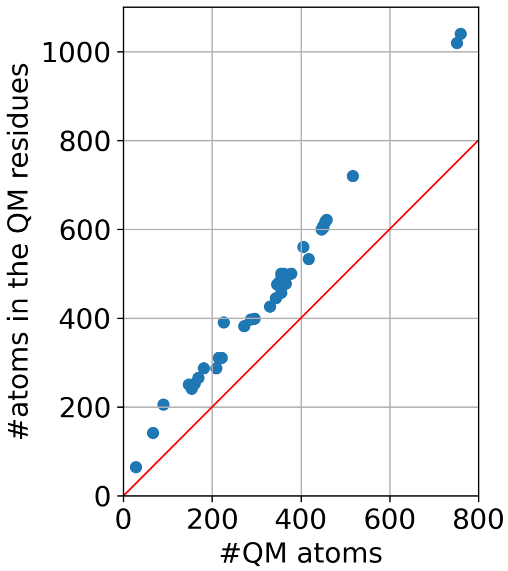

Figure S5: Number of atoms in the residue including QM atoms vs. the number of selected QM atoms alone.

S3 LNO-CCSD(T)/CBS Results

Table S1: $E^{X}_{\text{N-T}} - E^{X}_{\text{Normal}}$ corrections in kcal/mol for LNO-CCSD(T) calculations.

basis (X)	QM atoms	**TS1**	**INT**	**TS2**	**PS**
cc-pVTZ	101	-0.28	-0.37	-0.43	-0.15
aug-cc-pVTZ	101	-0.07	0.00	-0.07	-0.01
cc-pVTZ	238	-0.28	-0.19	-0.04	0.07

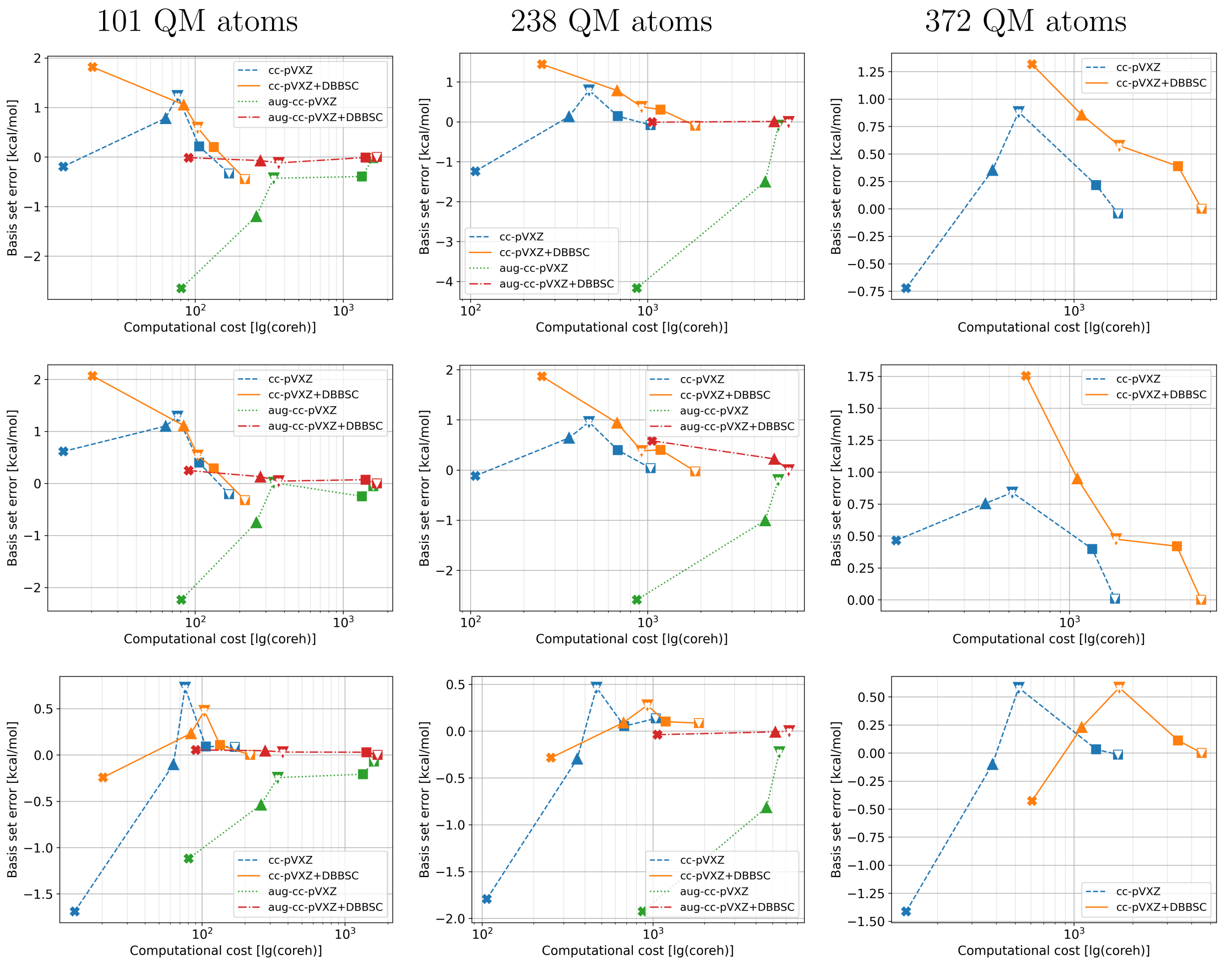

Figure S6: Convergence of basis set errors of LNO-CCSD(T) energies (from top to bottom: **INT**, **TS2** and **PS**) with computational cost for three QM regions: 101, 238 and 372 atoms from left to right. The basis set errors are measured compared to the best converged CBS(T,Q) reference. DBBSC stands for the density-based basis set correction. Runtime for CBS data points are the sum of the corresponding two calculations.

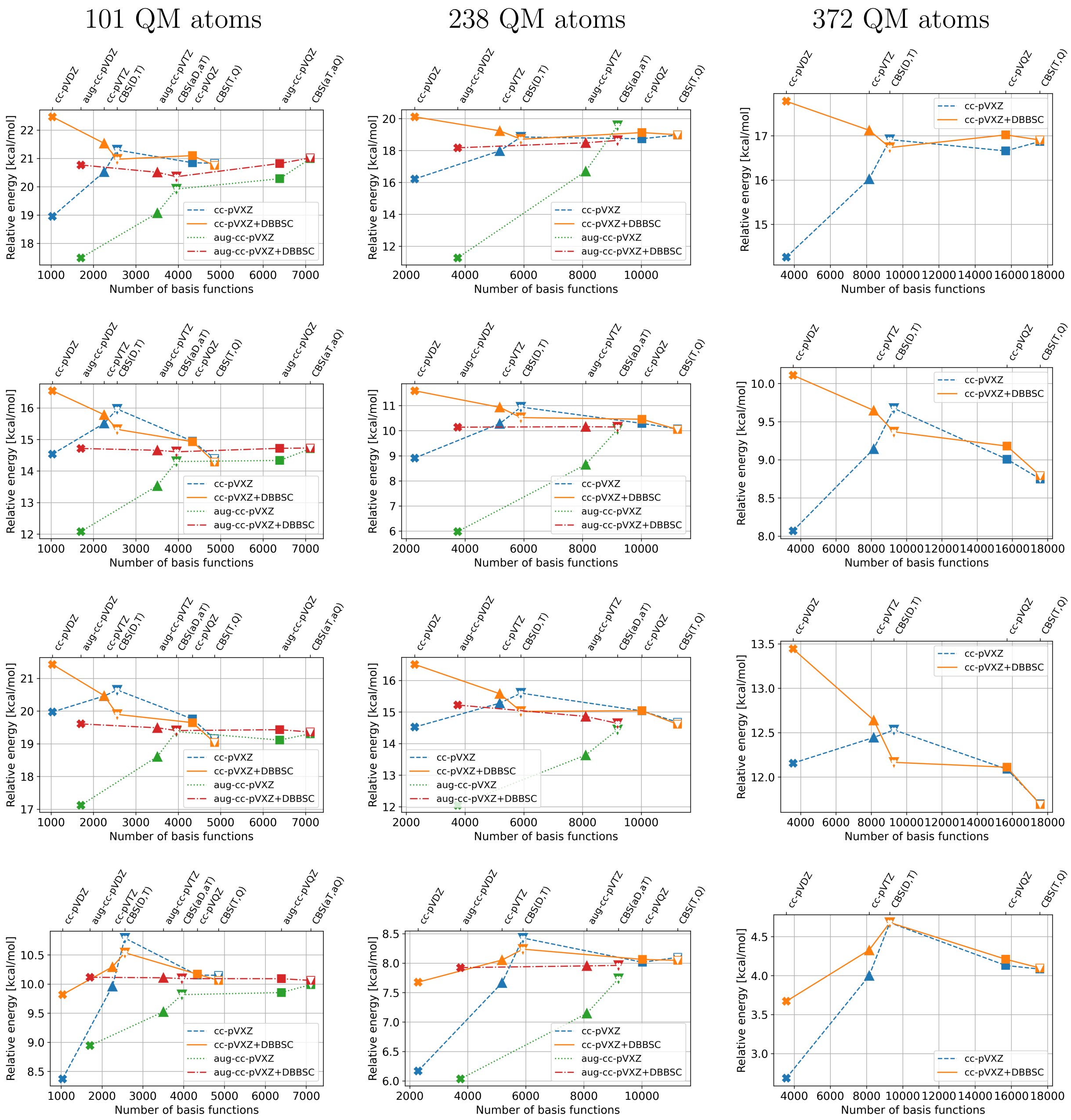

Figure S7: Convergence of relative energies (from top to bottom: **T1**, **INT**, **TS2** and **PS**) with size of basis sets for three QM regions: 101, 238 and 372 atoms from left to right. CBS($X, X+1$) refers to the CBS extrapolated values, 'a' denotes the use of diffuse basis sets. DBBSC stands for the density-based basis set correction. Extrapolated basis function counts are estimated as a 25% surplus of the previous step of the cardinal number.

S4 DFT Benchmark

Table S2: LNO-CCSD(T)/CBS references and DFT errors [kcal/mol] calculated with the 101 atom QM region with various functionals and cc-pVTZ basis set. D3 and D4 refer to the empirical dispersion correction.

method	**TS1–TS2**	**TS1**	**INT**	**TS2**	**PS**
$\Delta E^{\mathrm{CBS}(aT,aQ)}_{\text{N-T LNO-CCSD(T)}}$	1.66	20.95	14.72	19.29	10.05
BLYP-D3	-5.71	-6.91	-3.32	-1.20	-0.78
PW91-D3	-2.84	-5.10	-4.18	-2.26	-0.92
BP86-D3	-3.35	-7.09	-5.96	-3.74	-1.58
PBE-D3	-2.98	-5.62	-4.49	-2.64	-1.25
SCAN-D3	-1.83	-4.86	-4.42	-3.03	-1.72
M06-L-D3	-4.75	-5.12	-0.84	-0.36	-1.54
B97M-V[a]	-3.08	-2.27	0.68	0.81	-0.65
TPSS-D3	-3.29	-5.23	-3.48	-1.94	-0.56
TPSSh-D3	-2.46	-3.74	-2.59	-1.28	-0.46
PBE0-D3	-0.93	-2.02	-2.31	-1.09	-0.91
B3LYP-D3	-3.52	-3.80	-1.89	-0.28	-0.60
M06-2X-D3	-1.02	-2.16	-2.54	-1.14	-1.85
MN15-D3	-0.31	-2.24	-1.44	-1.93	-2.47
BHLYP-D3	-1.50	0.14	0.89	1.63	-0.01
ωB97X-D3	-2.87	-3.98	-1.89	-1.11	-1.11
ωB97M-V[a]	-2.22	-3.16	-1.29	-0.95	-1.27
LC-ωPBE-D3	-0.40	-1.04	-1.39	-0.63	-1.20
CAM-B3LYP-D3	-1.58	-2.12	-1.96	-0.54	-0.70
revDSDPBEP86-D4[b]	-1.54	-0.53	0.37	1.00	-0.21

[a] dispersion is calculated by the VV10 non-local density functional.
[b] D4 empirical dispersion is used.

S4.1 Density sensitivity

We use the density sensitivity measure (S^{DFT}) as

$$S^{\mathrm{DFT}} = E^{\mathrm{DFT}}[\rho^{\mathrm{LDA}}] - E^{\mathrm{DFT}}[\rho^{\mathrm{HF}}] \tag{1}$$

defined as the difference of energies evaluated at the LDA and HD densities.

Sensitivities in Table S3 are typical of the specific DFT rungs: semi-local functionals have S^{DFT}, especially for **TS1**. For equilibrium structures (**PS**), density sensitivity is significantly

smaller. Hybrid DFT methods and especially RSH functionals are the least affected by this issue, although the S^{DFT} values exhibit a sizable variation.

Table S3: Density sensitivities (S^{DFT}) [kcal/mol] of DFT functionals.

functional	**TS1**	**INT**	**TS2**	**PS**
BLYP	-3.55	-2.92	-2.51	-0.56
PW91	-4.07	-3.56	-3.23	-0.96
BP86	-4.19	-3.68	-3.36	-1.10
PBE	-3.97	-3.52	-3.23	-1.00
SCAN	-3.54	-3.13	-2.68	-0.93
M06-L	-2.01	-2.60	-2.22	-0.79
B97M-V	-1.71	-2.03	-1.71	-0.59
TPSS	-4.07	-3.58	-3.30	-0.99
TPSSh	-3.21	-2.93	-2.66	-0.78
PBE0	-1.88	-1.91	-1.66	-0.46
B3LYP	-1.86	-1.60	-1.24	-0.15
M06-2X	-0.14	-1.22	-0.09	-0.34
MN15	-1.37	-1.94	-2.10	-1.14
BHLYP	0.81	0.50	0.81	0.56
ωB97X	0.13	-0.61	-0.32	-0.18
ωB97M-V	-0.77	-1.11	-1.03	-0.52
LC-ωPBE	-0.65	-1.40	-1.09	-0.47
CAM-B3LYP	-0.80	-0.77	-0.48	-0.03

Table S4: Errors in relative energies [kcal/mol] calculated by selected DFT functionals with def2-TZVPPD basis set, against LNO-CCSD(T)/CBS references.

method	#atom	**TS1**	**INT**	**TS2**	**PS**
$E_{\text{N-T LNO-CCSD(T)}}^{\text{CBS}(aT,aQ)}$	101	20.9	14.7	19.3	10.0
PBE-D3	101	-5.2	-4.5	-2.6	-1.1
B3LYP-D3	101	-3.5	-1.9	-0.4	-0.4
PBE0-D3	101	-1.7	-2.4	-1.2	-0.8
ωB97X-D3	101	-3.6	-1.8	-1.1	-0.7
$E_{\text{N-T LNO-CCSD(T)}}^{\text{CBS}(T,Q)}$	238	18.7	9.9	14.6	8.1
PBE-D3	238	-4.5	-3.7	-1.8	-0.5
B3LYP-D3	238	-3.4	-1.5	-0.2	-0.7
PBE0-D3	238	-1.2	-1.5	-0.6	-0.5
ωB97X-D3	238	-4.2	-2.1	-1.7	-2.1
$E_{\text{Normal LNO-CCSD(T)}}^{\text{CBS}(T,Q)}$	372	16.9	8.8	11.7	4.1
PBE-D3	372	-4.6	-3.8	-1.8	-0.4
B3LYP-D3	372	-3.4	-1.2	0.5	0.0
PBE0-D3	372	-1.5	-1.5	-0.3	-0.1
ωB97X-D3	372	-3.9	-1.1	-0.3	-0.7

Table S5: Basis set dependence of the PBE0-D3 QM/MM energies for three QM regions. Energies are in kcal/mol.

basis	#atom	**TS1**	**INT**	**TS2**	**PS**
def2-SVP	101	14.1	9.7	16.7	8.3
def2-SVPD	101	15.7	10.2	16.9	9.0
def2-TZVPP	101	19.3	12.8	18.5	9.5
def2-TZVPPD	101	19.2	12.4	18.1	9.3
def2-QZVPPD	101	19.5	12.6	18.3	9.5
def2-SVP	238	11.7	4.8	11.9	6.1
def2-SVPD	238	13.7	5.9	12.3	6.7
def2-TZVPP	238	17.4	8.4	14.0	7.6
def2-TZVPPD	238	17.6	8.4	14.0	7.6
def2-SVP	372	10.5	5.0	10.6	3.5
def2-SVPD	372	12.1	5.2	10.4	3.8
def2-TZVP	372	15.3	7.4	11.6	4.1
def2-TZVPP	372	15.4	7.4	11.5	4.1
def2-TZVPPD	372	15.4	7.3	11.4	4.0

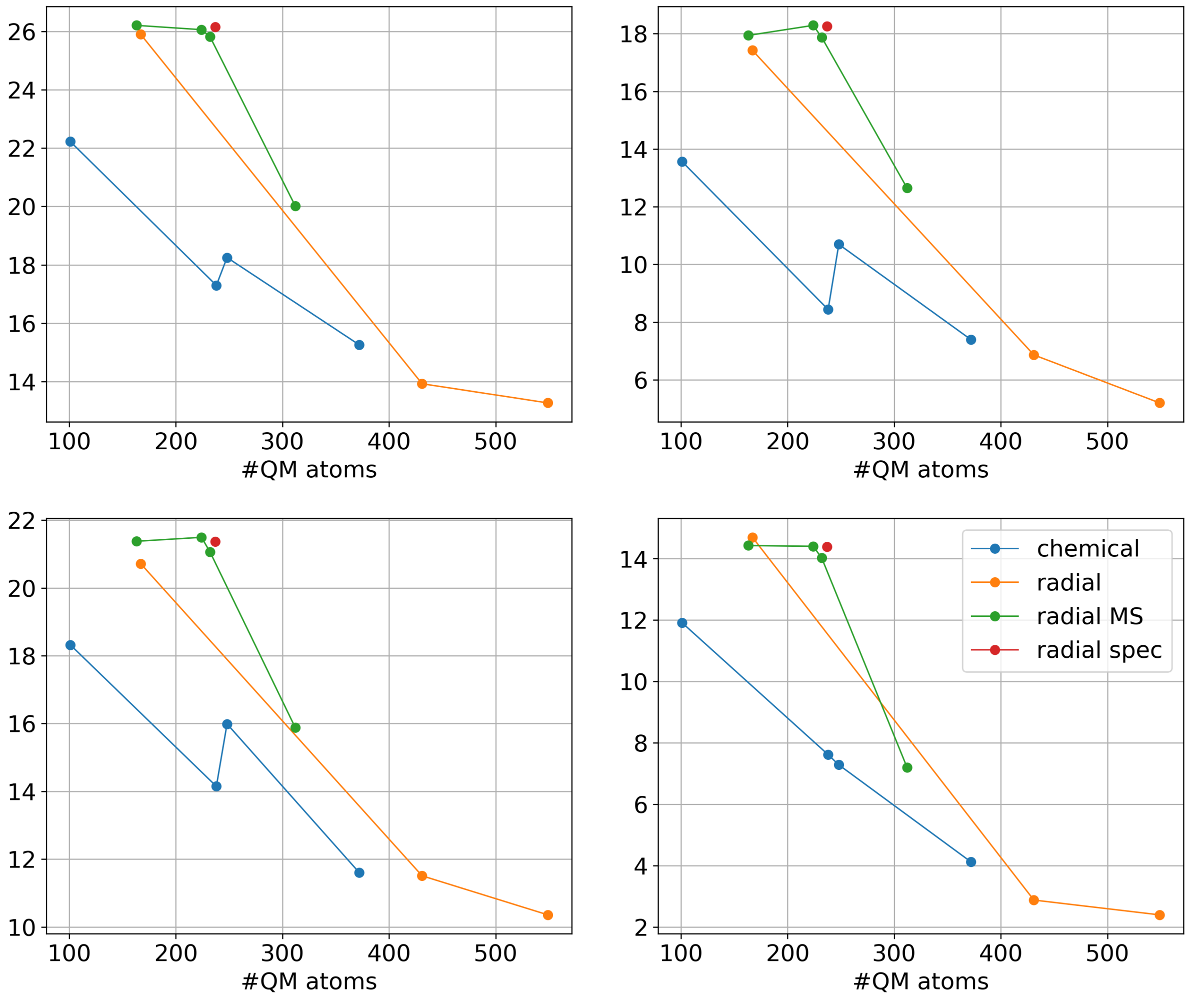

Figure S8: Convergence of the PBE0-D3/def2-TZVP/CHARMM36m relative energies (vs the reactants state RS) of the four other stationary points. **Needs labels: top row TS1, INT, bottom row: TS2, PS** Grouping needs consideration: **spec** means it is a modified version of another selection (e.g. a residue added or removed), **MS** means it was created based on multiple structures. In the main text, **spec** selections are omitted.

S5 Embedding

S5.1 Embedding Errors

Table S6: Huzinaga embedding errors [kcal/mol] with different active atom lists, calculated with PBE0-D3/def2-TZVP-in-PBE-D3/def2-SVP against the reference of full system energies at PBE0-D3/def2-TZVP.

#atoms					
active	total	TS1	INT	TS2	PS
17	238	0.14	0.15	0.45	0.42
28	238	-0.09	-0.03	0.35	0.43
35	238	0.00	-0.12	0.13	0.13
40	238	-0.35	-0.59	-0.24	-0.08
41	238	-0.16	-0.11	0.39	0.40
44	238	-0.20	-0.38	-0.01	0.04
81	238	-0.64	-0.65	-0.57	-0.57
28	372	0.16	0.07	0.35	0.46
35	372	0.29	0.18	0.38	0.34
40	372	-0.08	-0.34	-0.03	0.13
41	372	0.14	0.08	0.47	0.50
44	372	0.11	-0.05	0.26	0.28
81	372	-0.20	-0.13	-0.19	-0.43

S5.2 ONIOM Electrostatic Embedding

While for the MM atoms, only the charged defined in the force field can be used, the question rises how the atoms of the environment QM layers should be represented. Interestingly, charges derived from the electrostatic potential (CHELPG) based on the GGA density caused further convergence issues and thus proved ineffective.[1] Here, we used localization-based IAO charges, which works consistently, and similarly the MM charges defined in CHARMM36m produce equivalent results. Notably, other population analysis schemes (NBO,[2] RESP,[3] CMx[4]) could be employed for the electrostatic embedding of the inner ONIOM layer.

Table S7: ONIOM errors [kcal/mol] with different active atom lists within a 238-atom QM region, calculated with PBE0-D3/def2-TZVP inner and variable outer layer, against the reference of full system energies at PBE0-D3/def2-TZVP.

#active atoms	LL method	TS1	INT	TS2	PS
72	PBE-D3/def2-SVP	0.71	-1.15	-0.29	0.02
86	PBE-D3/def2-SVP	1.95	0.35	1.01	1.14
104	PBE-D3/def2-SVP	0.40	0.39	0.68	0.34
125	PBE-D3/def2-SVP	1.00	0.99	1.16	0.84
132	PBE-D3/def2-SVP	1.08	1.02	1.20	0.98
136	PBE-D3/def2-SVP	0.96	0.74	0.80	0.43
142	PBE-D3/def2-SVP	0.88	0.75	0.87	0.56
143	PBE-D3/def2-SVP	1.04	0.78	0.86	0.58
175	PBE-D3/def2-SVP	-0.49	-0.50	-0.46	-0.70
72	GFN2-xTB	-2.48	-7.11	-8.33	-5.97
86	GFN2-xTB	-0.69	-3.87	-4.54	-3.85
104	GFN2-xTB	0.15	-3.34	-3.85	-0.83
132	GFN2-xTB	-0.63	-2.92	-3.80	-2.42
136	GFN2-xTB	0.52	-1.52	-2.12	-0.76
142	GFN2-xTB	0.44	-1.85	-2.59	-0.84
143	GFN2-xTB	0.68	0.03	-0.56	-0.68
175	GFN2-xTB	0.76	-0.85	-0.95	0.39

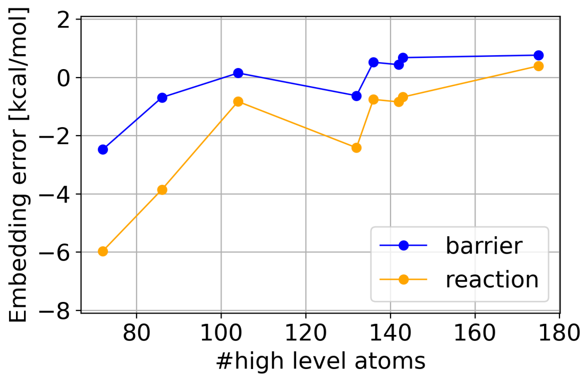

Figure S9: ONIOM embedding errors (vs. PBE0-D3/def2-TZVP results for the 238-atom QM region) [kcal/mol] using GFN2-xTB as environment method.

References

(1) Breneman, C. M.; Wiberg, K. B. Determining atom-centered monopoles from molecular electrostatic potentials. The need for high sampling density in formamide conformational analysis. *J. Comput. Chem.* **1990**, *11*, 361.

(2) Foster, J. P.; Weinhold, F. *J. Am. Chem. Soc.* **2004**, *102*, 7211.

(3) Bayly, C. I.; Cieplak, P.; Cornell, W.; Kollman, P. A. A well-behaved electrostatic potential based method using charge restraints for deriving atomic charges: the RESP model. *The Journal of Physical Chemistry* **1993**, *97*, 10269–10280.

(4) Marenich, A. V.; Jerome, S. V.; Cramer, C. J.; Truhlar, D. G. Charge Model 5: An Extension of Hirshfeld Population Analysis for the Accurate Description of Molecular Interactions in Gaseous and Condensed Phases. *Journal of Chemical Theory and Computation* **2012**, *8*, 527–541.
